# Targeted Degradation of STING by a Neutrophil Membrane‐Coated Nanoplatform Suppresses Microglial Pyroptosis After Subarachnoid Hemorrhage

**DOI:** 10.1002/advs.76553

**Published:** 2026-07-11

**Authors:** Ruotian Zhang, Kaikun Yuan, Haoyu Zou, Hanfeng Qin, Jiale Liu, Jianda Sun, Feifan Zhang, Yuankui Wang, Tong Wang, Jiaxing Dai, Chunxu Li, Yichi Chen, Yingfeng Tu, Huaizhang Shi

**Affiliations:** ^1^ Department of Neurosurgery The First Affiliated Hospital of Harbin Medical University Harbin China; ^2^ State Key Laboratory of Multi‐organ Injury Prevention and Treatment Guangdong Provincial Key Laboratory of New Drug Screening & Guangdong‐Hong Kong‐Macao Joint Laboratory for New Drug Screening School of Pharmaceutical Sciences Southern Medical University Guangzhou China; ^3^ Department of Ultrasound Harbin Medical University Cancer Hospital Harbin China

**Keywords:** biomimetic nanoparticles, microglial pyroptosis, PROTAC, STING, subarachnoid hemorrhage

## Abstract

Subarachnoid hemorrhage (SAH) is a life‐threatening cerebrovascular disease in which neuroinflammation and neuronal death critically contribute to poor outcomes. Here, we identify aberrant STING activation as a key driver of microglial pyroptosis and post‐SAH injury. Transcriptomic and biochemical analyses support a close association between STING signaling and microglial pyroptosis, with MAPK signaling acting as a functionally relevant downstream pathway. Based on this mechanistic insight, we engineered MG1 peptide‐functionalized, neutrophil membrane‐coated STING‐PROTAC nanoparticles (MG1@NM‐Px) to enable blood‐brain barrier penetration, microglia‐targeted delivery, and efficient STING degradation in vivo. This catalytic degradation suppressed the inflammasome‐related activation and GSDME‐associated pyroptotic signaling, reduced pro‐inflammatory cytokine secretion, and prevented neuronal apoptosis. Histopathological examination showed preserved Nissl body integrity, while behavioral testing revealed significant improvements in neurological function. Collectively, this engineered neutrophil membrane‐coated STING‐PROTAC nanoplatform effectively degrades STING, inhibits microglial pyroptosis, and provides robust neuroprotection in SAH. This work establishes a novel biomimetic nanomedicine strategy for SAH therapy and opens new avenues for treating neuroinflammation‐related disorders.

## Graphical abstract

MG1@NM‐Px serves as a microglia‐targeted STING‐degrading nanoplatform for subarachnoid hemorrhage. Following systemic administration, it crosses the blood–brain barrier and accumulates in activated microglia. STP1‐mediated STING ubiquitination and degradation suppress MAPK/inflammasome signaling, GSDME‐mediated pyroptosis, and IL‐1β release, revealing a targeted approach for mitigating neuroinflammation and neurological injury after SAH.

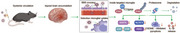

## 1 Introduction

Subarachnoid hemorrhage (SAH) is a devastating cerebrovascular emergency characterized by high mortality and profound neurological disability [1]. Growing evidence indicates that long‐term outcomes are determined not by the primary hemorrhage itself but by early brain injury (EBI) occurring within minutes to hours after onset [2]. EBI encompasses a series of acute pathological events—including blood–brain barrier (BBB) disruption, brain edema, oxidative stress, and neuroinflammation [3]—among which microglia‐driven inflammatory responses have emerged as central contributors to neuronal dysfunction and progressive neurological deterioration [4]. Consequently, delineating the molecular mechanisms underlying pathological microglial activation after SAH and developing effective strategies to restrain this inflammatory cascade represent critical unmet challenges in neurovascular research [5].

Among the diverse mechanisms that drive neuroinflammation, microglial pyroptosis has emerged as a critical amplifier of early brain injury [6]. Characterized by inflammasome activation and gasdermin‐mediated membrane pore formation, pyroptosis leads to rapid cellular rupture and the release of potent pro‐inflammatory cytokines such as IL‐1β and IL‐18 [7], thereby inducing cascading neuronal damage [8]. Although inhibition of the NLRP3/caspase‐1/gasdermin D axis has shown neuroprotective effects in ischemic stroke and traumatic brain injury [9], the upstream signals that trigger microglial pyroptosis in the context of SAH remain poorly defined [10]. The stimulator of interferon genes (STING), a central cytosolic DNA sensor, is recognized as a key driver of sterile inflammation [11]. Emerging evidence indicates that aberrant STING activation can promote NLRP3 inflammasome assembly and induce pyroptotic cell death [12]; however, whether and how STING contributes to microglial pyroptosis after SAH‐induced brain injury is entirely unknown. Moreover, the reported crosstalk between STING and mitogen‐activated protein kinase (MAPK) pathways [13] suggests a potential STING–MAPK axis linking innate immune sensing to inflammatory cell death, representing a previously overlooked pathological node in SAH‐related EBI.

Given that STING likely acts upstream of microglial pyroptosis after SAH, its inhibition offers a rational strategy to intercept the inflammatory amplification that drives EBI [14]. However, currently available STING inhibitors rely on transient occupancy of the active site and are unable to provide sustained pathway suppression within the rapidly initiated and progressively amplified inflammatory milieu that characterizes early SAH pathology [15]. In contrast, proteolysis‐targeting chimeras (PROTACs) induce complete and recurrent degradation of STING, thereby blocking the pathological activation of the MAPK pathway at its source and more effectively restraining microglial inflammatory responses and pyroptosis [16, 17]. A recently developed CRBN‐based STING degrader has demonstrated potent anti‐inflammatory activity in vivo, supporting the therapeutic potential of STING depletion in neuroinflammatory conditions [18]. Nevertheless, PROTAC molecules typically exhibit high molecular weight and poor lipophilicity, resulting in limited BBB penetration and insufficient cellular selectivity, which substantially restricts their application in central nervous system disorders [19]. Thus, to harness the full therapeutic potential of STING degradation for SAH‐induced EBI, an effective delivery system that can both traverse the BBB and selectively target activated microglia is urgently needed [20].

**SCHEME 1:**
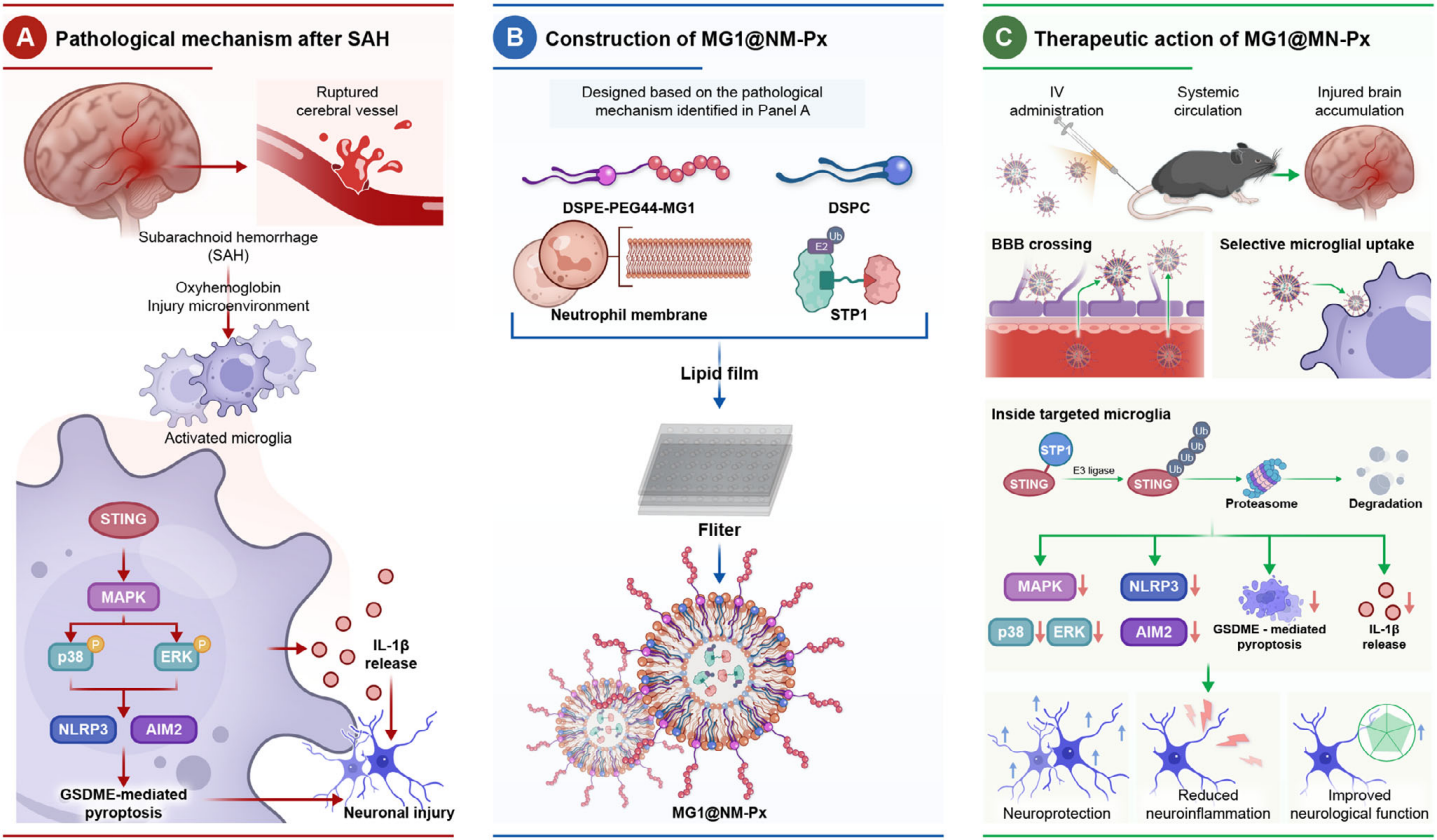
Schematic illustration of the pathological mechanism after SAH, construction of MG1@NM‐Px, and its therapeutic action in suppressing microglial pyroptosis. (A) Proposed pathological mechanism of STING‐associated microglial pyroptosis after subarachnoid hemorrhage (SAH). Following rupture of cerebral vessels, hemoglobin‐derived injury signals activate microglia and induce STING‐associated MAPK signaling, leading to inflammasome‐related activation, GSDME‐mediated pyroptosis, IL‐1β release, neuroinflammation, and neuronal injury. (B) Construction of the biomimetic STING‐degrading nanoplatform MG1@NM‐Px. STP1, a STING‐targeting PROTAC molecule, was incorporated into a liposomal formulation composed of DSPC and DSPE‐PEG44‐MG1, followed by neutrophil membrane coating to generate MG1@NM‐Px. (C) Therapeutic action of MG1@NM‐Px after intravenous administration. MG1@NM‐Px accumulates in the injured brain, crosses the BBB, and promotes microglia‐associated uptake. After intracellular delivery, STP1 induces ubiquitin–proteasome‐dependent STING degradation, thereby suppressing MAPK activation, inflammasome‐related signaling, GSDME‐mediated pyroptosis, and IL‐1β release, ultimately reducing neuroinflammation and improving neurological recovery after SAH.

To address the unresolved mechanisms underlying microglial pyroptosis during SAH‐induced early brain injury, we first investigated the upstream signaling events that drive this inflammatory cell death. Our study identifies STING as a critical mediator of microglial pyroptosis after SAH and supports a functional link between aberrant STING activation and MAPK signaling, which subsequently triggers inflammasome activation and gasdermin‐mediated pyroptotic injury [13, 21, 22]. This finding establishes a previously unrecognized STING–MAPK–pyroptosis signaling axis contributing to neuroinflammatory amplification during early brain injury. Based on this mechanistic insight, we further developed a targeted therapeutic strategy by selectively degrading STING using a PROTAC‐based approach [23, 24, 25]. To enable efficient brain delivery, we engineered a biomimetic nanoplatform (MG1@NM‐Px) by integrating neutrophil membrane camouflage with the microglia‐targeting peptide MG1 [26], thereby achieving inflammation‐homing capability, BBB penetration, and microglia‐specific uptake [27, 28]. To our knowledge, this study represents the first demonstration that targeted degradation of STING via PROTAC can effectively suppress microglial pyroptosis and mitigate SAH‐induced early brain injury, providing a mechanistically informed strategy for controlling neuroinflammation after SAH (Scheme 1).

## 2 Results and Discussion

### 2.1 STING is Upregulated After SAH and Contributes to Neurological Deficits, Neuroinflammation, and Neuronal Injury

**FIGURE 1:**
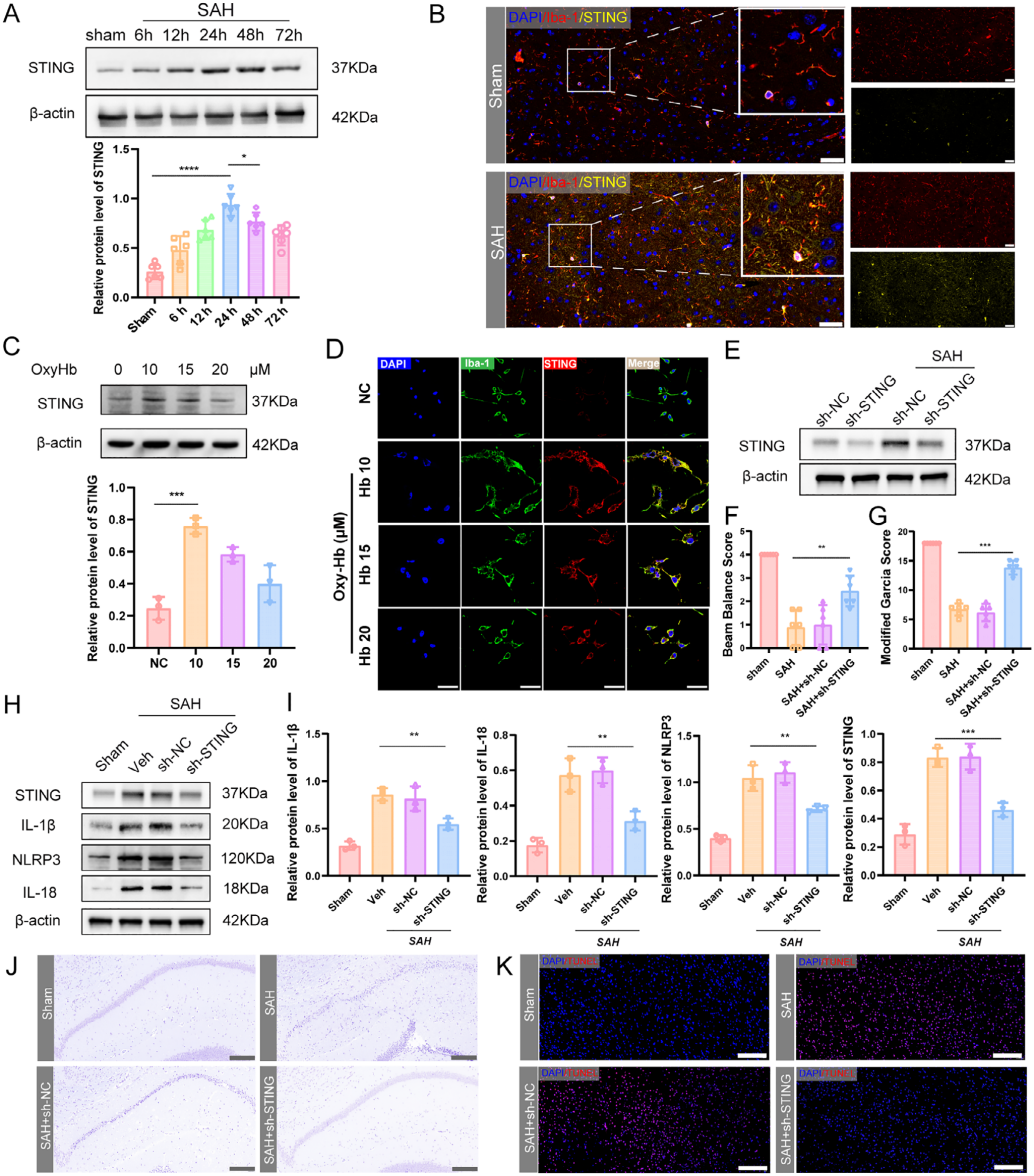
STING is upregulated after SAH and contributes to neurological deficits, neuroinflammation, and neuronal injury. (A) Western blot analysis showing a marked increase in STING protein levels in the ipsilateral cortex of SAH mice compared with sham controls. (B) Representative confocal laser scanning microscopy (CLSM) images demonstrating robust STING expression predominantly in Iba1^+^ microglia in the cortex after SAH. Scale bar: 40 µm. (C) Western blot analysis showing dose‐dependent upregulation of STING in BV‐2 microglial cells stimulated with increasing concentrations of OxyHb. (D) CLSM images illustrating enhanced cytoplasmic STING immunofluorescence in BV‐2 cells following OxyHb treatment. Scale bar: 40 µm. (E) Western blot confirming efficient microglia‐specific knockdown of STING in vivo using TMEM119‐driven AAV‐shSTING. (F) Beam balance test performed 24 h post‐SAH showing significant improvement in motor coordination in the sh‐STING group. (G) Modified Garcia neurological scores at 24 h post‐SAH demonstrating ameliorated neurological deficits in mice with STING knockdown. (H) Western blot analysis of inflammatory mediators (IL‐1β, NLRP3, IL‐18) showing marked upregulation after SAH and significant suppression following STING knockdown. (I) Quantification of Western blot band intensities for inflammatory markers, showing the relative protein expression across groups. (J) Representative Nissl staining images showing neuronal injury in the hippocampal region after SAH, which was markedly alleviated by STING knockdown. Scale bar: 200 µm. (K) Representative immunofluorescence images of TUNEL staining showing reduced apoptotic cell death in the sh‐STING group compared with SAH mice. TUNEL‐positive nuclei are shown in red; DAPI counterstaining in blue. Scale bar: 200 µm. For in vivo Western blot quantification in panel A, *n* = 6 mice per group; for in vitro Western blot quantification in panels C and E and inflammatory protein quantification in panel I, *n* = 3 independent biological replicates per group. For behavioral assessments in panels F and G, *n* = 6 mice per group. Quantitative analyses corresponding to immunofluorescence, Nissl staining, and TUNEL staining are provided in Figures S1 and S3. Statistical significance was determined by unpaired two‐tailed Student's *t*‐test for two‐group comparisons and one‐way ANOVA followed by Tukey's multiple‐comparison test for multiple‐group comparisons. *p < 0.05; **p < 0.01; ***p < 0.001; ns, not significant.

To delineate the temporal dynamics and functional relevance of STING signaling following subarachnoid hemorrhage (SAH), we first examined its expression in both in vivo and in vitro models. In the murine SAH model, STING protein levels in the ipsilateral cortex were markedly elevated compared with sham controls, reaching a peak at 24 h post‐injury and gradually declining thereafter (Figure 1A). This dynamic profile suggests a rapid activation of STING signaling during the acute phase of early brain injury. Immunofluorescence co‐staining further revealed that STING was predominantly localized to Iba1^+^ microglia in peri‐hemorrhagic regions (Figure 1B and Figure S1A), indicating that microglia represent the principal cellular source of STING activation after SAH. Consistent with the in vivo findings, exposure of BV‐2 microglial cells to oxyhemoglobin (OxyHb) significantly increased STING expression in vitro. Western blot analysis showed that STING protein levels reached a maximum after 24 h of stimulation with 10 µm OxyHb, while higher concentrations led to a mild decline; therefore, 10 µm was selected for subsequent experiments (Figure 1C). Immunofluorescence analysis revealed enhanced cytoplasmic STING staining in OxyHb‐treated BV‐2 cells, further confirming STING activation under hemoglobin‐derived stress (Figure 1D and Figure S1B). Collectively, these results demonstrate that STING expression is markedly elevated both in the ipsilateral cortex of SAH mice and in OxyHb‐stimulated microglia, suggesting a critical role for STING in mediating SAH‐induced neuroinflammation.

To elucidate the role of STING in early brain injury (EBI), we next employed a microglia‐specific AAV‐mediated knockdown strategy. The viral construct contained the microglia‐specific TMEM119 promoter and an EGFP reporter, and was injected intracerebroventricularly three weeks prior to SAH induction. Western blotting and immunofluorescence confirmed efficient STING knockdown in brain tissue (Figure 1E and Figures S1C and S2), ensuring that the observed effects arose from modulating central innate immune cells rather than astrocytes or infiltrating peripheral immune cells.

Neurological performance was assessed 24 h after SAH using the beam balance test and modified Garcia score. In the beam balance test, SAH mice showed marked motor and coordination impairment compared with the sham group, whereas STING knockdown significantly improved performance, with scores increasing from 0.89 ± 0.75 in the SAH group to 2.44 ± 0.66 in the sh‐STING group (Figure 1F). Consistently, modified Garcia scores were profoundly reduced after SAH (6.67 ± 1.03) compared with the sham group (18.00 ± 0.00), while STING knockdown markedly improved neurological function (13.83 ± 1.17) (Figure 1G). These findings indicate that aberrant STING activation contributes to neurological deficits after SAH, while its inhibition ameliorates motor and coordination impairments.

We further examined inflammatory mediators in the brain. Western blot analysis revealed substantial upregulation of IL‐1β, NLRP3, and IL‐18 following SAH, all of which were markedly suppressed by STING knockdown (Figure 1H,I). As a central cytosolic DNA sensor, STING promotes inflammasome assembly and cytokine maturation. Our findings align with this mechanism, demonstrating that STING functions as an upstream initiator of microglial inflammatory cascades after SAH. By suppressing STING, the amplification of neuroinflammation can be effectively interrupted.

Neuronal morphology and survival were assessed by Nissl and TUNEL staining. SAH resulted in pronounced neuronal shrinkage, nuclear condensation, and fragmentation, whereas these pathological changes were markedly alleviated in the sh‐STING group (Figure 1J and Figure S3A). Consistently, STING knockdown significantly reduced the number of TUNEL‐positive cells after SAH (Figure 1K and Figure S3B). Together with the reduced inflammatory mediator expression observed above, these findings indicate that STING knockdown attenuates SAH‐induced neuroinflammation‐associated neuronal injury. These results support STING as an upstream regulator of inflammatory injury during the acute phase of SAH‐induced early brain injury.

Taken together, these data demonstrate that STING is robustly upregulated after SAH, predominantly in microglia, and serves as a key driver of neuroinflammation, neuronal injury, and neurological deficits. STING knockdown effectively mitigates these pathological processes, supporting STING as a critical molecular target in SAH‐induced early brain injury.

### 2.2 STING Regulates Microglial Pyroptosis During the Acute Phase of SAH

**FIGURE 2:**
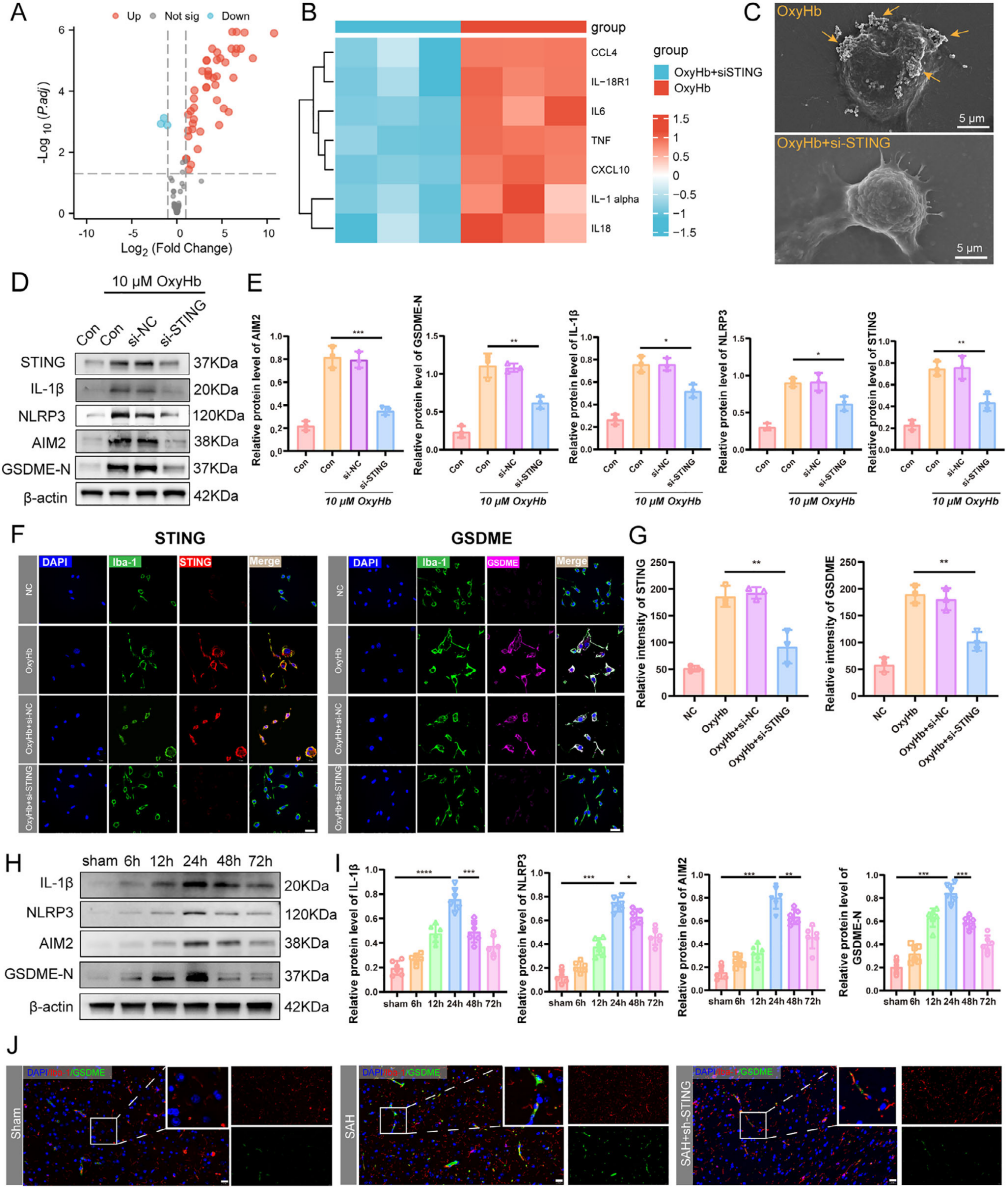
STING activation drives microglial pyroptosis in vitro and in vivo. (A,B) Olink proteomics showing increased inflammatory and pyroptosis‐related cytokines in OxyHb‐treated BV‐2 cells, which were reduced by STING knockdown. (C) Scanning electron microscopy images illustrating pyroptotic ultrastructure (membrane rupture, vesicle‐like protrusions) in OxyHb‐treated BV‐2 cells, largely absent after STING knockdown. Scale bar: 5 µm. (D,E) Western blot and quantification of IL‐1β, NLRP3, AIM2, and cleaved GSDME‐N in BV‐2 cells under the indicated treatments. (F,G) Immunofluorescence staining and semi‐quantification showing co‐upregulation of STING and GSDME after OxyHb stimulation, attenuated by STING knockdown. Green: Iba1; Red: STING; Magenta: GSDME; Blue: DAPI. Scale bar: 10 µm. (H&I) Time‐dependent increases of pyroptosis‐associated proteins in the ipsilateral cortex after SAH with corresponding quantification. *n* = 6 (J) Immunofluorescence images showing elevated GSDME in Iba1^+^ microglia after SAH, which was reduced by STING knockdown. Green: GSDME; Red: Iba1; Blue: DAPI. Scale bar: 20 µm. Quantitative data are presented as mean ± SEM. For Olink proteomic analysis in panels A and B, *n* = 3 independent biological replicates per group. For in vitro Western blot quantification in panels D and E, *n* = 3 independent biological replicates per group. For immunofluorescence quantification in panels F and G, *n* = 3 independent biological replicates per group, with at least three randomly selected fields analyzed for each replicate. For in vivo Western blot quantification in panels H and I, n = 6 mice per group. Quantitative analysis corresponding to panel J is provided in Figure S6. Statistical significance was determined by one‐way ANOVA followed by Tukey's multiple‐comparison test. *p < 0.05; **p < 0.01; ***p < 0.001; ns, not significant.

Having established that STING is rapidly upregulated after SAH and that microglia‐specific STING silencing markedly alleviates early brain injury—manifested by improved neurological scores, reduced inflammatory mediators, and attenuated neuronal loss—we next sought to define the downstream effector program linking STING activation to neuroinflammation and tissue damage. To this end, we performed Olink‐based proteomic profiling in OxyHb‐stimulated BV‐2 microglia with or without STING knockdown. Compared with the OxyHb+si‐STING group, STING‐competent microglia exhibited a pronounced increase in multiple pro‐inflammatory cytokines, including IL‐1β, IL‐6, and TNF‐α (Figure 2A), indicating that STING amplifies the inflammatory output of hemoglobin‐derived stress. Notably, several of the most prominently altered factors (e.g., CCL4, IL‐18, IL‐6, and IL‐1α) [29] are tightly linked to inflammasome activation and pyroptotic signaling (Figure 2B), prompting the hypothesis that STING aggravates SAH pathology by promoting microglial pyroptosis.

Scanning electron microscopy (SEM) imaging supported this hypothesis by demonstrating classical pyroptotic morphology in OxyHb‐treated BV‐2 cells, including cell swelling, membrane disruption, and vesicle‐like protrusions [30]. These abnormalities were largely absent in the OxyHb+si‐STING group, where cells maintained smooth and intact membranes (Figure 2C). This morphological evidence indicates that STING silencing protects microglia against OxyHb‐induced pyroptotic injury. To further validate pyroptosis‐associated membrane damage, we performed PI/Hoechst 33342 double staining and LDH release assays as complementary functional analyses. OxyHb stimulation markedly increased PI‐positive BV‐2 cells and LDH release, indicating enhanced membrane permeability and impaired membrane integrity, whereas STING knockdown substantially attenuated both effects (Figure S4). In addition, high‐magnification SEM images further confirmed prominent membrane ballooning, membrane disruption, and pyroptotic body‐like structures in OxyHb‐treated cells, which were reduced after STING knockdown (Figure S5). These complementary functional and morphological data provide more direct evidence that STING contributes to OxyHb‐induced pyroptosis‐associated membrane damage in microglia. Consistent with these observations, Western blot analysis revealed significant increases in NLRP3, AIM2, and cleaved GSDME‐N following OxyHb treatment, whereas STING‐deficient cells exhibited markedly reduced expression of these pyroptosis‐related proteins (Figure 2D,E). Immunofluorescence staining further demonstrated co‐upregulation of STING and GSDME in OxyHb‐treated microglia, which was significantly attenuated after STING knockdown (Figure 2F,G). Together, these data confirm that STING activation enhances OxyHb‐induced microglial pyroptosis.

We next examined whether similar mechanisms occur in vivo. In the ipsilateral cortex of SAH mice, IL‐1β, NLRP3, AIM2, and GSDME‐N levels were markedly elevated, peaking at 24 h after hemorrhage (Figure 2H,I), indicating prominent pyroptotic activation during the acute phase of SAH. Immunofluorescence analysis confirmed extensive GSDME induction in Iba1^+^ microglia, which was substantially reduced following STING knockdown (Figure 2J and Figure S6). These results collectively demonstrate that microglial pyroptosis is strongly activated after SAH and is directly regulated by STING. We further examined GSDMD expression in both in vivo and in vitro models (Figure S7). Unlike the robust activation of GSDME, GSDMD showed no prominent or consistent alteration across the SAH time course or after Hb/OxyHb stimulation in microglia. This difference may reflect distinct gasdermin‐executor engagement in the present SAH‐related microglial injury context: GSDMD is classically activated downstream of inflammasome‐associated inflammatory caspases [31], whereas GSDME can be cleaved by caspase‐3 and convert apoptosis‐associated signaling into lytic pyroptosis‐like cell death [32, 33]. These findings support GSDME‐associated signaling as the more prominent pyroptotic pathway in this study. Mechanistically, these findings are consistent with previous reports showing that STING activation promotes inflammasome assembly and gasdermin‐mediated pyroptotic signaling, thereby driving inflammatory cell death [34]. Our results provide direct evidence that microglial pyroptosis represents a downstream effector mechanism of STING hyperactivation.

Overall, our findings identify aberrant STING activation as a pivotal upstream driver of inflammasome assembly and GSDME cleavage, thereby promoting microglial pyroptosis and amplifying secondary neuroinflammatory injury. Inhibition of STING effectively interrupts this positive feedback loop, providing a mechanistic foundation for its neuroprotective role in early brain injury following SAH.

### 2.3 STING Regulates BV‐2 Microglial Pyroptosis in Association With MAPK Signaling

**FIGURE 3:**
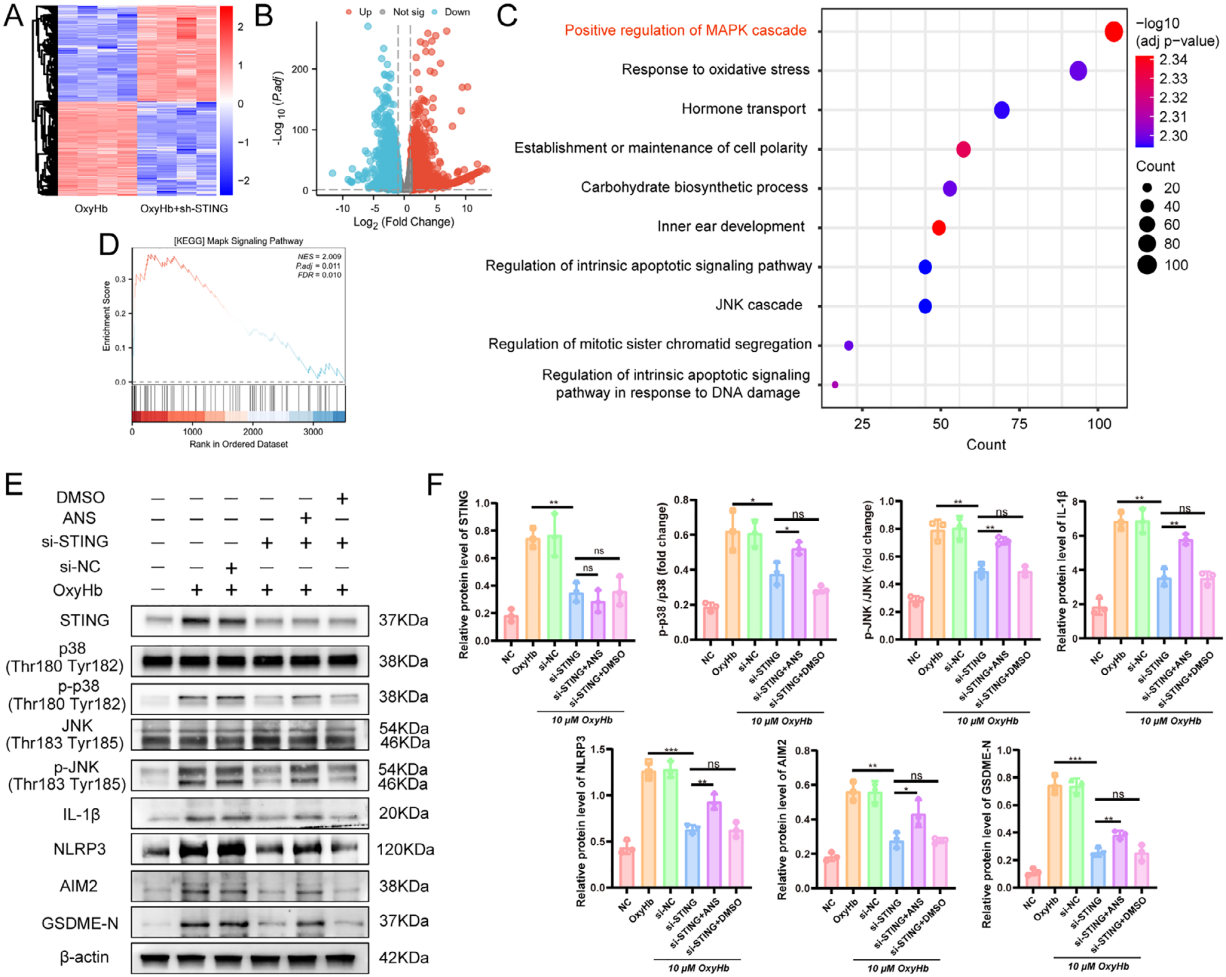
STING is functionally linked to MAPK signaling during microglial pyroptosis. (A,B) Volcano plot and heatmap showing differentially expressed genes between OxyHb‐treated and OxyHb+shSTING BV‐2 cells. (C) GO enrichment revealing significant enrichment in MAPK‐related biological processes. (D) GSEA analysis showing robust enrichment of the MAPK signaling pathway. (E) Western blot analysis of STING, phosphorylated MAPKs (p‐p38, p‐ERK), and pyroptosis‐related proteins (NLRP3, AIM2, cleaved GSDME‐N) in BV‐2 cells under indicated treatments, including ANS rescue. (F) Densitometric quantification of protein expression. Quantitative data are presented as mean ± SEM. For RNA‐seq analysis in panels A–D, *n* = 3 independent biological replicates per group. For Western blot quantification in panels E and F, *n* = 3 independent biological replicates per group. Statistical significance was determined by one‐way ANOVA followed by Tukey's multiple‐comparison test. *p < 0.05; **p < 0.01; ***p < 0.001; ns, not significant.

To further elucidate the molecular mechanism by which STING regulates microglial pyroptosis, we performed transcriptomic sequencing of BV‐2 cells from the OxyHb and OxyHb+shSTING groups. A total of 4039 differentially expressed genes (DEGs) were identified, including 1997 upregulated and 2042 downregulated genes (Figure 3A,B). Gene Ontology (GO) enrichment analysis revealed that these DEGs were predominantly associated with biological processes such as positive regulation of the MAPK cascade, response to oxidative stress, and establishment or maintenance of cell polarity (Figure 3C). In line with this, gene set enrichment analysis (GSEA) demonstrated a significant enrichment of the MAPK signaling pathway (Figure 3D). These findings suggest that MAPK signaling is functionally associated with STING‐dependent microglial pyroptosis under SAH‐related oxidative stress.

Previous studies have shown that the MAPK family—including p38, JNK, and ERK—plays a central role in coordinating inflammatory and cell‐death signaling [35]. In inflammatory injury contexts, MAPK phosphorylation promotes NLRP3 inflammasome assembly and caspase‐1 activation, subsequently driving GSDMD/GSDME cleavage and pyroptosis [36]. Conversely, pyroptosis‐related cytokines such as IL‐1β, IL‐18, and HMGB1 can reactivate the MAPK pathway, forming a self‐amplifying inflammatory loop that exacerbates tissue injury [7]. Thus, MAPK signaling acts both as an upstream regulator and a downstream amplifier of pyroptotic inflammation. Based on these findings, we hypothesized that STING is functionally linked to MAPK‐associated microglial pyroptosis after SAH.

To verify this hypothesis, we examined the activation states of key MAPK components. Western blot analysis showed that OxyHb stimulation markedly increased STING expression, and enhanced phosphorylation of p38 and ERK, accompanied by elevated levels of the pyroptosis‐related proteins NLRP3, AIM2, and cleaved GSDME‐N (Figure 3E,F). In contrast, STING‐silenced BV‐2 cells failed to exhibit increases in p‐p38 and p‐ERK after OxyHb exposure, and the induction of pyroptosis‐associated proteins was similarly diminished. Notably, treatment with the MAPK activator anisomycin (ANS) restored the phosphorylation of p38 and ERK in STING‐knockdown cells and reinstated the expression of NLRP3, AIM2, and GSDME‐N (Figure 3E,F and Figure S8). These findings support that STING is functionally required for robust MAPK activation associated with OxyHb‐induced microglial pyroptosis.

Collectively, these data support a functional link between STING and MAPK signaling in microglial pyroptosis under SAH‐related oxidative stress. OxyHb‐induced phosphorylation of p38 and ERK, together with downstream pyroptosis‐associated signaling, was attenuated by STING knockdown and partially restored by anisomycin, supporting MAPK as a functionally relevant downstream pathway. Although these transcriptomic, biochemical, and rescue data support this association, additional pathway‐dissection studies will be valuable to further define the precise upstream–downstream relationship. Guided by this mechanistic insight, we next developed MG1@NM‐Px, a biomimetic STING‐degrading nanoplatform designed for efficient brain delivery and microglia‐targeted intervention, thereby translating the identified STING‐associated pathogenic mechanism into a therapeutic strategy for suppressing microglial pyroptosis after SAH.

### 2.4 Mechanism‐Guided Construction of MG1@NM‐Px Enables Targeted STING Degradation in Microglia

Our previous findings established STING as a central driver of microglial hyperactivation and pyroptotic injury during SAH‐induced early brain injury (EBI), highlighting its therapeutic relevance. However, the poor BBB permeability and inadequate cellular selectivity of conventional STING‐PROTAC molecules impede their utility in the CNS [37]. To overcome these limitations, we developed a biomimetic nanoplatform—MG1@NM‐Px—comprising neutrophil membrane camouflage and MG1 peptide functionalization to enable inflammation‐homing, microglia‐specific targeting, and efficient intraparenchymal delivery of STING degraders.

To construct MG1@NM‐Px, STP1 was prepared according to a previously reported protocol (Figure S9) [18]. STP1 is a reported modular STING‐targeting PROTAC containing a STING‐recognition unit, a linker, and an E3‐ligase‐recruiting moiety. It induces ubiquitin–proteasome‐dependent STING degradation rather than transient pathway inhibition. To overcome the delivery limitations of free PROTAC molecules in vivo, STP1 was encapsulated into the MG1@NM‐Px biomimetic nanoplatform for brain delivery and microglia‐targeted intracellular STING degradation. The synthetic route, ^1^H NMR spectrum, HPLC chromatogram, and LC‐MS/HRMS spectrum of STP1 are shown in Figures S9–S12.

**FIGURE 4:**
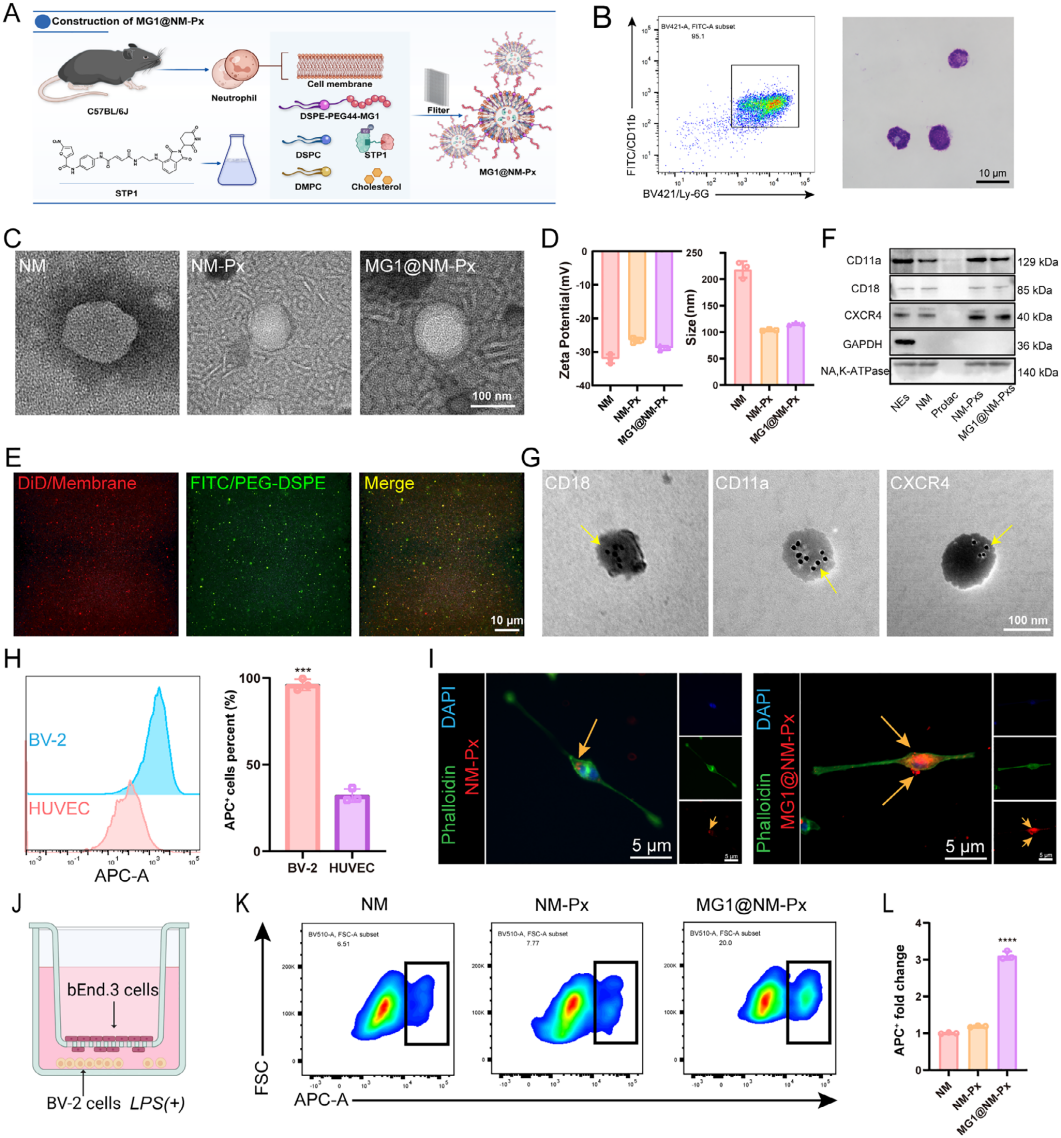
Preparation, characterization, and targeting capability of MG1@NM‐Px nanoparticles. (A) Schematic illustration of the fabrication process of MG1@NM‐Px nanoparticles. (B) Characterization of isolated neutrophils by Ly6G/CD11b flow cytometry and Giemsa staining. (C) TEM images of NM‐NPs, NM‐Px NPs, and MG1@NM‐Px NPs showing uniform spherical morphology. (D) Hydrodynamic size and ζ‐potential measurements of NM‐NPs, NM‐Px NPs, and MG1@NM‐Px NPs. (E) Colocalization imaging of neutrophil membranes (DiD‐labeled, red) and DSPE‐PEG‐FITC (green) after co‐extrusion, confirming successful surface modification. (F) Western blot analysis demonstrating the retention of neutrophil membrane proteins (CD11a, CD18, CXCR4) in NM‐coated formulations. (G) Immunogold TEM images of MG1@NM‐Px labeled with anti‐CD11a, anti‐CD18, or anti‐CXCR4 antibodies, confirming the preservation of key membrane proteins on the nanoparticle surface. (H) Flow cytometric quantification of differential nanoparticle uptake in BV‐2 microglia vs. HUVECs. (I) CLSM visualization of MG1@NM‐Px internalization in BV‐2 cells. Blue: DAPI; Green: Phalloidin (F‐actin); Red: MG1@NM‐Px. (J) Transwell‐based in vitro BBB penetration assay of NM, NM‐Px, and MG1@NM‐Px. (K) Flow cytometric analysis and (L) quantitative comparison of nanoparticle uptake by BV‐2 cells after BBB traversal. Quantitative data are presented as mean ± SEM. For hydrodynamic size and ζ‐potential measurements in panel D, *n* = 3 independent measurements per group. For flow cytometric quantification in panels H and L, *n* = 3 independent biological replicates per group. For two‐group comparisons, statistical significance was determined by unpaired two‐tailed Student's *t*‐test. For multiple‐group comparisons, statistical significance was determined by one‐way ANOVA followed by Tukey's multiple‐comparison test. *p < 0.05; **p < 0.01; ***p < 0.001; ns, not significant.

Subsequently, DSPE‐PEG‐MG1, STP1 liposomes, and neutrophil membranes were uniformly mixed and co‐extruded through a polycarbonate membrane to generate MG1@NM‐Px nanoparticles (Figure 4A). Murine neutrophils were isolated from bone marrow and purified via density‐gradient centrifugation. Flow cytometric analysis using Ly6G/CD11b co‐staining confirmed a purity exceeding 95%, verifying the robustness of the isolation procedure (Figure 4B). Giemsa staining further confirmed neutrophil identity, revealing multilobed nuclei and azurophilic granules characteristic of mature neutrophils (Figure 4B). Neutrophil membranes (NM) were isolated from mouse bone marrow and purified by density gradient centrifugation. Flow cytometric analysis with Ly6G/CD11b co‐staining and Giemsa staining showed a purity exceeding 95% and revealed multilobed nuclei and azurophilic granule characteristics of mature neutrophils, demonstrating successful isolation of neutrophils and the robustness of the separation procedure (Figure 4B).

Purified neutrophils were next subjected to membrane extraction. TEM imaging revealed intact, continuous lipid bilayer structures with uniform morphology, confirming successful acquisition of structurally preserved neutrophil membranes (Figure 4C). TEM analysis demonstrated that both NM‐Px NPs (MG1@NM‐Px without MG1 peptide) and MG1@NM‐Px NPs formed uniform spherical nanostructures (Figure 4C). Dynamic light scattering revealed that native NMs possessed an average diameter of 218.43 ± 15.70 nm. After co‐extrusion, NM‐NPs and MG1@NM‐Px exhibited reduced diameters of 103.93 ± 1.50 nm and 115.33 ± 1.80 nm, respectively (Figure 4D). The modest increase in MG1@NM‐Px size is consistent with MG1 peptide surface modification. Both nanoparticle formulations displayed negatively charged ζ‐potentials (−26.53 ± 0.75 mV for NM‐NPs and −28.87 ± 0.65 mV for MG1@NM‐Px), comparable to that of native NMs (−32.07 ± 1.35 mV), confirming preservation of membrane‐surface electrochemical properties (Figure 4D). Coomassie brilliant blue staining further showed that MG1@NM‐Px retained a protein‐band profile similar to that of the neutrophil membrane fraction, supporting successful membrane coating and membrane‐protein preservation (Figure S13). A flow cytometry‐based endothelial association assay further confirmed the functional contribution of the neutrophil membrane coating. In TNF‐α‐activated bEnd.3 endothelial cells, both DiD‐labeled NM‐Px and MG1@NM‐Px showed markedly higher relative MFI than uncoated Lipo‐Px, indicating enhanced endothelial association mediated by the neutrophil membrane coating (Figure S14). Notably, MG1 functionalization did not compromise this membrane‐associated interaction.

To verify surface modification, FITC‐labeled PEG‐DSPE (green) and DiD‐labeled neutrophil membranes (red) were co‐extruded, yielding strong yellow fluorescence signals attributable to colocalization, confirming efficient incorporation of MG1‐PEG‐DSPE onto the NM surface (Figure 4E). To determine whether NM‐NPs and MG1@NM‐Px NPs retained key chemotactic and adhesion‐related membrane proteins from native NMs, we first performed Western blot analysis. As shown in Figure 4F, both nanoparticle formulations preserved the expression of CD11a, CD18, and CXCR4, indicating successful transfer of functional membrane proteins during the coating process. Given the superior spatial resolution and high labeling specificity of colloidal gold–conjugated antibody TEM imaging, we further visualized the localization of these proteins on the nanoparticle surface at the ultrastructural level. MG1@NM‐Px NPs were incubated with gold‐labeled anti‐CD11a, anti‐CD18, or anti‐CXCR4 antibodies, followed by TEM observation. As shown in Figure 4G, electron‐dense gold nanoparticles (highlighted by yellow arrows) were uniformly distributed along the surface of MG1@NM‐Px NPs, confirming that these chemotactic and adhesion‐associated membrane proteins were successfully retained on the outer layer of the biomimetic nanocarriers. In addition to preserving key membrane‐associated proteins, the nanoplatform also achieved efficient STP1 loading, with HPLC‐based quantification further confirmed successful STP1 incorporation into the liposomal formulation, with an average STP1 content of 40.8 µg/mg nanoparticles, corresponding to a DLC of 4.08 wt% and an EE of 7.18% (Figure S15). The in vitro release profile of STP1 from MG1@NM‐Px was further evaluated under physiological and acidic conditions. MG1@NM‐Px showed limited STP1 release in PBS at pH 7.4, with cumulative release of 10.54% ± 0.69% at 24 h and 14.97% ± 2.05% at 48 h. By contrast, acidic conditions at pH 5.5 markedly accelerated STP1 release, reaching 33.08% ± 3.74% at 24 h and 81.93% ± 2.06% at 48 h (Figure S16). These results suggest that MG1@NM‐Px limits premature STP1 leakage under physiological conditions while promoting PROTAC release in acidic intracellular compartments after cellular uptake. The colloidal stability of MG1@NM‐Px was further evaluated under physiological conditions. During incubation in PBS at 37°C for 7 days, MG1@NM‐Px maintained a stable hydrodynamic diameter and zeta potential, with no obvious size increase or surface charge fluctuation. SEM imaging at day 0 and 7 further showed preserved particle morphology without obvious aggregation (Figure S17). These results support the structural integrity and colloidal stability of the liposomal biomimetic nanoplatform under physiological conditions.

Next, we evaluated MG1‐mediated microglial targeting. Flow cytometry revealed significantly higher uptake of MG1@NM‐Px by BV‐2 microglia compared to HUVEC endothelial cells, with BV‐2 uptake reaching approximately threefold higher at 6 h (Figure 4H). This demonstrates strong microglia specificity and minimal nonspecific internalization. Immunofluorescence imaging corroborated these findings: MG1@NM‐Px showed intense intracellular red fluorescence extensively colocalizing with phalloidin‐labeled actin filaments in BV‐2 cells, whereas NM‐Px exhibited weak internalization (Figure 4I), confirming enhanced microglial binding and uptake mediated by MG1 functionalization. To evaluate BBB traversal, a bEnd.3–BV‐2 coculture transwell model was established. TEER values stabilized near 180 Ω·cm^2^, confirming an intact barrier suitable for permeability testing (Figure 4J). After exposure to DiD‐labeled nanoparticles, BV‐2 cells in the lower chamber displayed significantly higher fluorescence when treated with MG1@NM‐Px compared with NM‐Px. Quantification indicated more than a twofold increase in microglial uptake (Figure 4K,L), demonstrating that MG1@NM‐Px possesses both enhanced BBB‐crossing capability and robust microglial targeting.

Together, these results establish MG1@NM‐Px as a biomimetic nanoplatform capable of highly efficient BBB penetration, selective microglial accumulation, and optimized delivery of intracellular STING degraders—addressing key translational limitations of current PROTAC‐mediated CNS therapies.

### 2.5 Engineered Neutrophil‐Membrane‐Coated MG1@NM‐Px Nanoparticles Efficiently Degrade STING and Sustainably Suppress Microglial Pyroptotic Signaling

**FIGURE 5:**
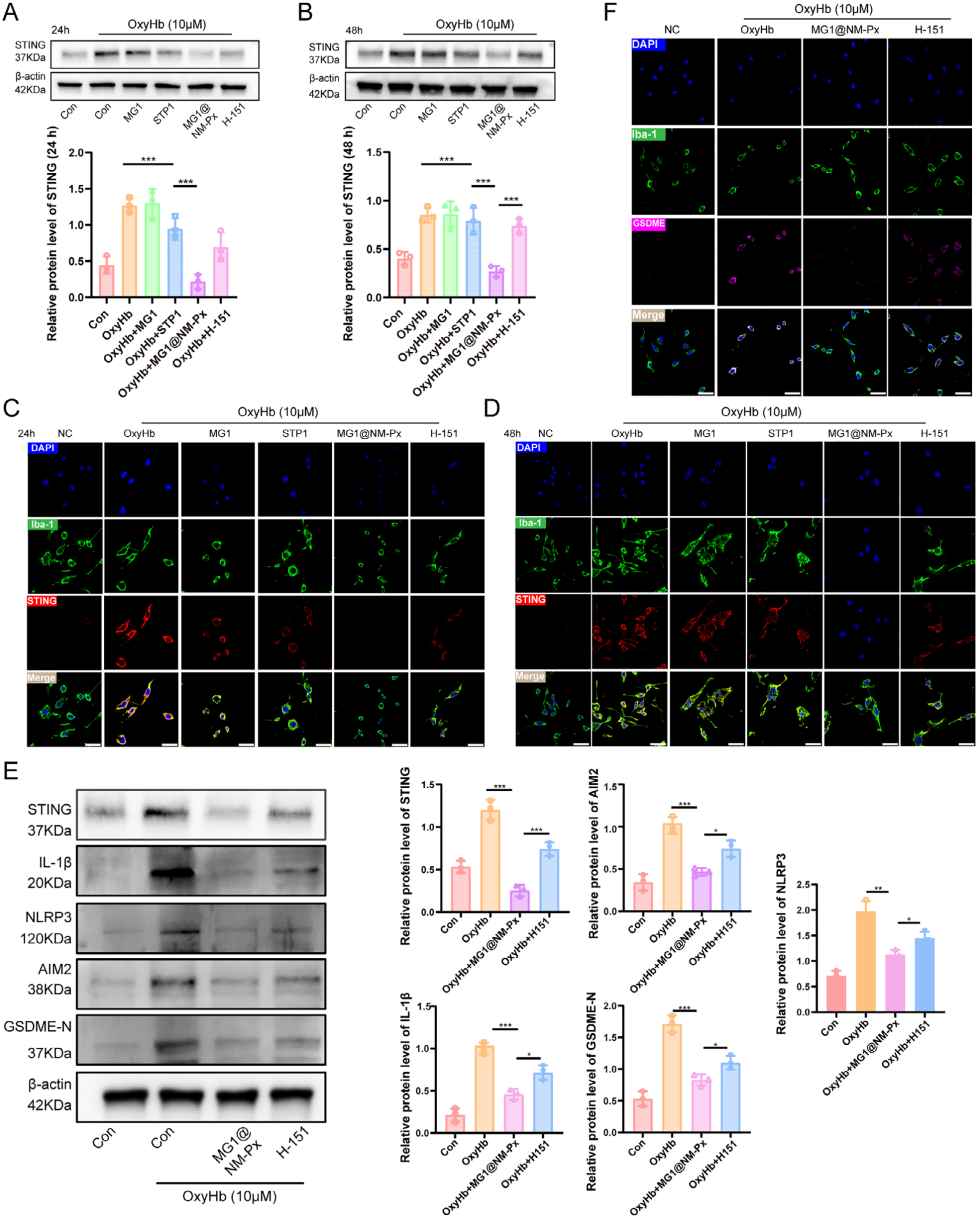
MG1@NM‐Px nanoparticles efficiently degrade STING and suppress STING‐dependent pyroptotic signaling in microglia (A) Western blot analysis of STING expression in BV‐2 microglial cells treated with OxyHb (10 µM) for 24 and 48 h, followed by different interventions (MG1, PROTAC, MG1@NM‐Px, or H‐151). (B) Quantification of STING protein levels at 24 and 48 h. MG1@NM‐Px shows the most potent and sustained STING degradation. Data are presented as mean ± SEM. *p* < 0.05, *p* < 0.01, *p* < 0.001. (C,D) Representative CLSM images at 24 (C) and 48 h (D) showing colocalization of Iba‐1 (green) and STING (red) in BV‐2 cells. MG1@NM‐Px markedly reduces intracellular STING fluorescence, whereas H‐151 shows only partial inhibition. Scale bar: 5 µm. (E) Western blot analysis of pyroptosis‐associated proteins (IL‐1β, NLRP3, AIM2, and cleaved GSDME‐N) after 48 h of OxyHb stimulation with or without MG1@NM‐Px or H‐151 treatment. Corresponding densitometric quantification is shown to the right. (F) Immunofluorescence staining of GSDME activation in BV‐2 cells after 48 h. MG1@NM‐Px effectively suppresses OxyHb‐induced GSDME upregulation, restoring fluorescence intensity to near‐baseline levels. Scale bar: 20 µm. Quantitative data are presented as mean ± SEM. For Western blot quantification in panels B and E, *n* = 3 independent biological replicates per group. For immunofluorescence quantification corresponding to panels C, D, and F, *n* = 3 independent biological replicates per group, with at least three randomly selected fields analyzed for each replicate; the corresponding quantitative results are provided in Figure S18. Statistical significance was determined by one‐way ANOVA followed by Tukey's multiple‐comparison test for comparisons among different treatment groups at each time point. *p < 0.05; **p < 0.01; ***p < 0.001; ns, not significant.

We next evaluated whether MG1@NM‐Px could effectively induce STING degradation in activated microglia. Prior to the comparative experiments, a dose–response analysis was performed to determine the optimal working concentration of MG1@NM‐Px. As shown in Figure S18, 10 µg/mL achieved robust STING reduction without detectable cytotoxicity and was therefore selected for subsequent studies. For in vitro comparison, H‐151 was used at 5 µm as a reference STING inhibitor, with the concentration selected based on previously published microglia‐related studies [38]. Consistent with OxyHb‐induced STING activation, OxyHb stimulation markedly elevated STING protein levels in BV‐2 cells (Figure 5A). Treatment with MG1@NM‐Px substantially reduced STING abundance. Quantitative analysis further demonstrated that MG1@NM‐Px produced significantly greater STING depletion than either free PROTAC or H‐151, a covalent small‐molecule inhibitor that blocks STING palmitoylation and activation, at 24 h (Figure 5B). Importantly, whereas STING expression partially rebounded in the H‐151 group at 48 h, MG1@NM‐Px maintained stable and sustained suppression, indicating prolonged intracellular degradation activity.

These findings were corroborated by immunofluorescence imaging (Figure 5C,D). In OxyHb‐treated cells, the microglial marker Iba‐1 (green) showed extensive cytoplasmic colocalization with STING (red), confirming robust STING upregulation. In contrast, MG1@NM‐Px markedly reduced STING fluorescence at both 24 h and 48 h, with signals nearly undetectable. H‐151 produced only partial fluorescence reduction and failed to prevent STING re‐accumulation at 48 h (Figure S19A,B). Together, these results indicate that MG1@NM‐Px enters microglia through MG1‐mediated targeting and achieves potent and sustained intracellular STING degradation through STP1 release. To further determine whether MG1@NM‐Px can suppress STING‐dependent pyroptotic signaling, we analyzed inflammasome and pyroptosis‐related proteins at the 48‐h treatment time point (Figure 5E). OxyHb stimulation for 48 h markedly increased the expression of NLRP3, AIM2, IL‐1β, and cleaved GSDME‐N, indicating robust activation of STING‐driven pyroptosis. In contrast, MG1@NM‐Px significantly reduced all four markers at the same time point, demonstrating markedly stronger inhibitory efficacy than H‐151. Consistent with these biochemical findings, immunofluorescence staining at 48 h (Figure 5F and Figure S19C) showed pronounced GSDME activation following OxyHb exposure, whereas MG1@NM‐Px effectively reversed this increase and restored GSDME fluorescence to near‐baseline levels.

Collectively, these findings demonstrate that MG1@NM‐Px mediates efficient and persistent STING degradation in microglia, thereby suppressing inflammasome activation and pyroptotic signaling. This sustained target depletion likely underlies its superior anti‐inflammatory efficacy compared with pharmacological STING inhibition.

### 2.6 In Vivo Brain Delivery, Microglial Targeting, and Therapeutic Efficacy of MG1@NM‐Px in SAH

**FIGURE 6:**
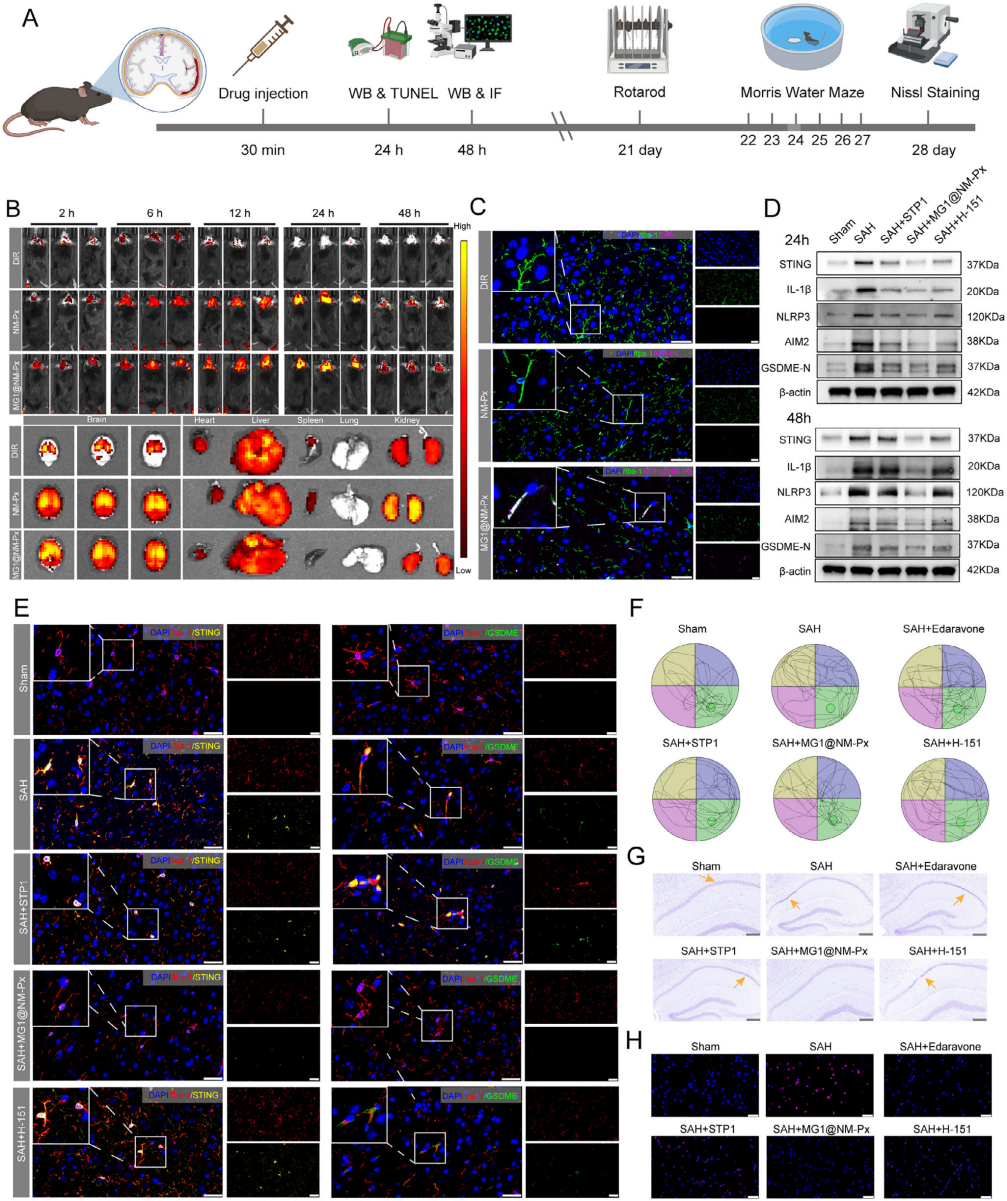
In vivo brain delivery, microglial targeting, and therapeutic efficacy of MG1@NM‐Px in SAH mice. (A) Experimental timeline illustrating SAH induction, nanoparticle administration, and subsequent molecular, histological, and behavioral assessments in C57BL/6 mice. (B) In vivo fluorescence imaging of mice receiving DiR, NM‐Px, or MG1@NM‐Px at 2, 6, 12, 24, and 48 h after administration. Representative ex vivo fluorescence images of the brain and major organs (heart, liver, spleen, lung, and kidney) collected at 24 h are shown below. (C) Representative confocal images showing brain tissue sections with nanoparticle distribution. MG1@NM‐Px exhibits increased localization within microglial regions compared with NM‐Px and DiR controls. Scale bar: 40 µm. Green: Iba‐1, Red: NPs with DiR. (D) Western blot analysis of STING and pyroptosis‐related proteins (IL‐1β, NLRP3, AIM2, and GSDME‐N) in ipsilateral cortex at 24 and 48 h post‐treatment across different therapeutic groups. (E) Representative immunofluorescence images of brain sections showing co‐localization of microglial marker Iba‐1 with STING (left panels) and GSDME (right panels) across treatment groups. Insets indicate magnified regions. Scale bar: 40 µm. (F) Representative swimming trajectories from the Morris water maze probe test demonstrating spatial memory performance, *n* = 6. Data are presented as mean ± SEM. (G) Nissl staining of hippocampal neurons showing neuronal morphology and survival following different treatments. Scale bar: 200 µm. (H) Representative TUNEL staining images indicating neuronal apoptosis in brain sections. Scale bar: 40 µm. Quantitative data are presented as mean ± SEM. For in vivo fluorescence imaging in panel B, *n* = 3 mice per group. For microglia‐associated localization analysis corresponding to panel C, *n* = 3 mice per group, with at least three randomly selected fields analyzed for each mouse. For Western blot analysis in panel D, *n* = 6 independent biological replicates per group. For immunofluorescence quantification corresponding to panel E, *n* = 3 mice per group, with at least three randomly selected fields analyzed for each mouse. For Morris water maze analysis corresponding to panel F and Figure S24, *n* = 6 mice per group. For Nissl staining in panel G, *n* = 3 mice per group. For TUNEL staining in panel H, *n* = 3 mice per group. Statistical significance was determined by one‐way ANOVA followed by Tukey's multiple‐comparison test. *p < 0.05; **p < 0.01; ***p < 0.001; ns, not significant.

Given that our transcriptomic analysis identified MAPK signaling as a key downstream effector of STING and our in vitro data established the STING–MAPK–pyroptosis axis as a central driver of microglial injury, we next asked whether targeted STING degradation by MG1@NM‐Px could achieve effective brain delivery and translate this mechanistic insight into in vivo therapeutic benefit in SAH. SAH was induced in male C57BL/6J mice by endovascular perforation, as previously described [39]. Briefly, a 5‐0 nylon filament was advanced through the carotid artery to perforate the anterior cerebral artery–middle cerebral artery bifurcation, while sham mice underwent the same procedure without perforation. As outlined in the experimental scheme (Figure 6A), mice subjected to SAH received intravenous administration of the indicated formulations 30 min after injury, with H‐151 included as a pharmacological STING‐inhibition comparator [40], followed by (i) longitudinal in vivo imaging to quantify brain accumulation during the early distribution window, (ii) molecular analyses at 24 and 48 h to evaluate STING degradation and downstream pyroptotic signaling, and (iii) behavioral testing during the recovery phase (rotarod at day 21 and Morris water maze from days 22 to 27) with terminal histopathological assessment (Nissl staining at day 28). To examine in vivo brain delivery and systemic biodistribution, fluorescence imaging was performed using DiR‐labeled nanocarriers. Three groups were compared: free DiR, NM‐Px, and MG1@NM‐Px. Longitudinal in vivo imaging showed that both NM‐Px and MG1@NM‐Px generated clear cranial fluorescence signals after intravenous administration, whereas free DiR showed much weaker brain‐associated fluorescence (Figure 6B). Quantitative analysis further confirmed that NM‐Px and MG1@NM‐Px exhibited comparable time‐dependent brain accumulation, and both were markedly higher than free DiR during the observation window (Figure S20A). This result suggests that the neutrophil membrane coating, which is shared by NM‐Px and MG1@NM‐Px, contributes substantially to inflammatory lesion homing and BBB traversal after SAH. Ex vivo imaging and quantitative fluorescence analysis of major organs, including the brain, heart, liver, spleen, lung, and kidney, further revealed the organ‐level biodistribution profiles of the nanocarriers (Figure 6B and Figure S20B). These whole‐brain and organ‐level fluorescence data demonstrate efficient brain‐associated accumulation of neutrophil membrane‐coated nanocarriers but do not distinguish their cellular localization within the brain parenchyma.

To further define the pharmacokinetic properties of the nanoplatform at the drug level, we quantified STP1 concentrations in blood after intravenous administration of MG1@NM‐Px. MG1@NM‐Px was administered at an STP1‐equivalent dose of 8.6 µg per 20 g mouse, corresponding to 0.43 mg/kg. STP1 rapidly reached peak blood concentration after injection, with a Cmax of 0.162 ± 0.008 mg/L at 5.0 min, followed by a gradual decline over 48 h (Figure S21). Non‐compartmental pharmacokinetic analysis showed an AUC0–t of 19.37 ± 0.29 mg/L·min, an AUC0–∞ of 19.86 ± 0.34 mg/L·min, a terminal half‐life of 537.18 ± 31.17 min, a CLz of 0.0217 ± 0.0004 L/min/kg, and an MRT0–t of 563.93 ± 6.23 min (Table S1). We further evaluated the brain‐to‐blood distribution of STP1 at 48 h after administration. The STP1 concentration in brain tissue was 4.73 ± 1.03 ng/g tissue, whereas the blood concentration was 0.63 ± 0.08 ng/mL, corresponding to a brain‐to‐blood ratio of 7.35 ± 0.74 mL/g (Table S2). These pharmacokinetic and tissue‐distribution data provide quantitative information on the systemic exposure and brain‐to‐blood distribution of STP1 after MG1@NM‐Px administration.

We next assessed cellular localization in brain sections. Immunofluorescence analysis showed that both NM‐Px and MG1@NM‐Px were detectable in the brain parenchyma, whereas MG1@NM‐Px exhibited markedly enhanced colocalization with Iba1^+^ microglia compared with NM‐Px (Figure 6C and Figure S22). To further determine whether this enhancement was attributable to MG1 functionalization, we performed a competitive blocking experiment in the SAH mouse model. DiR‐labeled MG1@NM‐Px showed substantial colocalization with Iba1‐positive microglia, whereas pretreatment with free MG1 markedly attenuated this signal. Semi‐quantitative analysis confirmed significant reductions in both the colocalization coefficient and microglia‐associated fluorescence intensity after MG1 blocking (Figure S23). These findings support that MG1 contributes, at least in part, to the enhanced microglial localization of MG1@NM‐Px in vivo. Thus, although both NM‐Px and MG1@NM‐Px exhibited comparable whole‐brain accumulation, MG1 functionalization more specifically improved microglia‐level targeting within the brain, consistent with the intended microglial target of STING degradation.

Consistent with the transcriptomic prediction that STING‐driven injury is coupled to pyroptosis‐related inflammatory signaling, MG1@NM‐Px produced the most pronounced reduction in STING protein in the ipsilateral cortex at both 24 h and 48 h, accompanied by marked suppression of IL‐1β, NLRP3, AIM2, and GSDME‐N (Figure 6D and Figure S24). These in vivo findings align with our in vitro mechanistic data showing that targeted STING degradation interrupts the downstream pyroptotic program triggered after hemorrhagic injury. In contrast, free STP1 showed limited efficacy, likely due to insufficient delivery efficiency, while H‐151 only partially attenuated downstream signaling without achieving comparable target depletion. Consistently, immunofluorescence analysis of brain tissues at 48 h further confirmed that MG1@NM‐Px markedly reduced STING and GSDME signals in microglial regions compared with the other treatment groups (Figure 6E and Figure S25).

We next evaluated whether these molecular effects translated into functional recovery. Spatial learning and memory were assessed using the Morris water maze. During the acquisition phase, SAH mice exhibited significantly prolonged escape latencies compared with sham controls (76.50 ± 2.30 s vs. 40.61 ± 5.67 s), indicating impaired spatial learning. MG1@NM‐Px markedly reduced the escape latency to 48.81 ± 4.92 s, whereas Edaravone and STP1 produced moderate improvements (62.11 ± 3.24 s and 64.38 ± 3.30 s, respectively). H‐151 showed limited improvement (75.97 ± 2.03 s) (Figure S26A). Swimming speed remained comparable among all groups, ranging from 18.53 ± 0.54 to 20.32 ± 0.42 cm/s, suggesting that the observed differences in escape latency were not caused by gross motor dysfunction (Figure S26B). In the probe trial, SAH markedly reduced the time spent in the target quadrant compared with sham controls (18.56 ± 2.57 s vs. 49.92 ± 2.81 s). MG1@NM‐Px significantly increased target‐quadrant occupancy to 40.73 ± 1.82 s, whereas Edaravone and STP1 showed moderate effects (30.97 ± 2.12 s and 29.71 ± 2.22 s, respectively), and H‐151 showed only limited improvement (19.56 ± 2.22 s) (Figure S26C). Consistently, platform crossings were reduced in SAH mice compared with sham controls (1.00 ± 0.52 vs. 8.33 ± 0.33), while MG1@NM‐Px restored platform crossings to 6.67 ± 0.76. Edaravone, STP1, and H‐151 produced only partial recovery, with 3.33 ± 0.49, 3.67 ± 0.56, and 2.67 ± 0.42 crossings, respectively (Figure 6F and Figure S26D).

Histological analyses further supported neuroprotection. Nissl staining revealed extensive neuronal loss and degenerative morphology in SAH mice, whereas MG1@NM‐Px preserved neuronal architecture (Figure 6G and Figure S27A). Consistently, TUNEL staining demonstrated a marked reduction in neuronal cell death following MG1@NM‐Px treatment (Figure 6H and Figure S27B). Histological examination of major organs (heart, liver, spleen, lung, and kidney) revealed no detectable structural abnormalities, indicating good in vivo biocompatibility (Figure S28), further evaluate systemic biosafety, blood routine and serum biochemical analyses were performed after treatment. No obvious abnormalities were observed in major hematological parameters or liver/kidney function‐related biochemical indices, supporting the preliminary systemic safety of MG1@NM‐Px under the current treatment conditions (Figure S29). Given the essential role of STING in innate host defense, particularly antiviral immunity, the potential immune‐related risks of STING degradation should also be considered. Although MG1@NM‐Px was designed for lesion‐enriched and microglia‐associated delivery, systemic exposure and possible impairment of host immune function cannot be completely excluded. Therefore, while our added hematological, biochemical, and histological analyses support favorable preliminary biosafety, future studies should further evaluate antiviral immune competence, immune‐cell profiling, and long‐term infection susceptibility after STING‐degrading therapy.

Collectively, these results show that MG1@NM‐Px not only enables efficient brain delivery and microglial targeting in vivo, but also functionally validates our earlier transcriptomic and mechanistic findings by demonstrating that STING‐directed intervention can suppress pyroptosis‐associated inflammatory injury and improve neurological recovery after SAH.

## 3 Conclusion

In conclusion, our study identifies aberrant activation of the STING pathway as a central molecular driver of early brain injury following subarachnoid hemorrhage (SAH). We demonstrate that STING is markedly upregulated in microglia during the acute phase of SAH and promotes neuroinflammation and neuronal injury through functionally associated MAPK signaling, thereby facilitating inflammasome assembly and GSDME‐mediated pyroptosis. Genetic or pharmacological suppression of STING effectively interrupts this MAPK‐dependent inflammatory cascade, attenuates neuronal loss, and improves neurological outcomes, establishing the STING–MAPK–pyroptosis axis as a key mechanistic pathway in SAH pathophysiology.

Building upon this mechanistic insight, we developed a neutrophil‐membrane–camouflaged PROTAC nanoplatform, MG1@NM‐Px, capable of selectively targeting microglia and catalytically degrading intracellular STING. Compared with conventional inhibitor‐based approaches, MG1@NM‐Px achieved sustained STING depletion and more effectively suppressed downstream pyroptotic signaling, resulting in robust neuroprotective effects at molecular, histological, and functional levels. Notably, this study represents, to our knowledge, the first application of PROTAC‐mediated targeted protein degradation for the treatment of SAH, providing proof‐of‐concept that degradation of innate immune signaling proteins can be harnessed to modulate neuroinflammation in hemorrhagic brain injury.

Collectively, our findings uncover a previously unrecognized STING–MAPK–driven microglial pyroptosis axis in SAH and establish targeted STING degradation as a promising therapeutic strategy. Beyond advancing the mechanistic understanding of neuroimmune regulation after hemorrhagic stroke, this work highlights the broader translational potential of PROTAC‐based nanomedicine for precision intervention in central nervous system inflammatory disorders.

## Author Contributions

R.Z. contributed to conceptualization, methodology, investigation, visualization, and writing –original draft preparation. K.Y., H.Z., and H.Q. contributed to methodology and investigation. J.L., J.S., and F.Z. contributed to formal analysis and data curation. Y.W., T.W., J.D., and C.L. contributed to software and validation. Y.C. contributed to conceptualization, supervision, and writing – review and editing. Y.T. contributed to supervision, project administration, and writing – review and editing. H.S. contributed to conceptualization, methodology, supervision, project administration, funding acquisition, and writing – review and editing. All authors reviewed and approved the final manuscript.

## Conflicts of Interest

The authors declare no conflict of interest.

## Supporting information




**Supporting File**: advs76553‐sup‐0001‐SuppMat.docx

## Data Availability

The data that support the findings of this study are available in the supplementary material of this article.
