# Supplementary material for: Targeted Degradation of STING by a Neutrophil Membrane‐Coated Nanoplatform Suppresses Microglial Pyroptosis After Subarachnoid Hemorrhage
Source: Adv Sci (Weinh). 2026 Jul 11:e76553. Online ahead of print. doi: 10.1002/advs.76553 (PMC13355893; doi:10.1002/advs.76553)
Supplement: Supplementary file 1 — Supporting File: advs76553‐sup‐0001‐SuppMat.docx [file ADVS-9999-e76553-s001.docx]

Supplementary Materials for

**Targeted degradation of STING by a neutrophil membrane-coated nanoplatform suppresses microglial pyroptosis after subarachnoid hemorrhage**

*Ruotian Zhang^1, #, *^, Kaikun Yuan^1, #^, Haoyu Zou^1, #^, Hanfeng Qin^2, #^, Jiale Liu^1^, Jianda Sun^1^, Feifan Zhang^1^, Yuankui Wang^1^, Tong Wang^1^, Jiaxing Dai^1^, Chunxu Li^1^, Yichi Chen^3, *^, Yingfeng Tu^2, *^, and Huaizhang Shi^1, *^*

^1^ Department of Neurosurgery, The First Affiliated Hospital of Harbin Medical University, Harbin 150001, China.

^2^ State Key Laboratory of Multi-organ Injury Prevention and Treatment, Guangdong Provincial Key Laboratory of New Drug Screening & Guangdong-Hong Kong-Macao Joint Laboratory for New Drug Screening, School of Pharmaceutical Sciences, Southern Medical University, Guangzhou 510515, China

^3^ Department of Ultrasound, Harbin Medical University Cancer Hospital, Harbin 150001, China.

^#^ These authors contributed equally to this work.

*Correspondence should be addressed to: huaizhangshi@163.com; tuyingfeng1@smu.edu.cn; zhangruotian@hrbmu.edu.cn; 202001382@hrbmu.edu.cn.


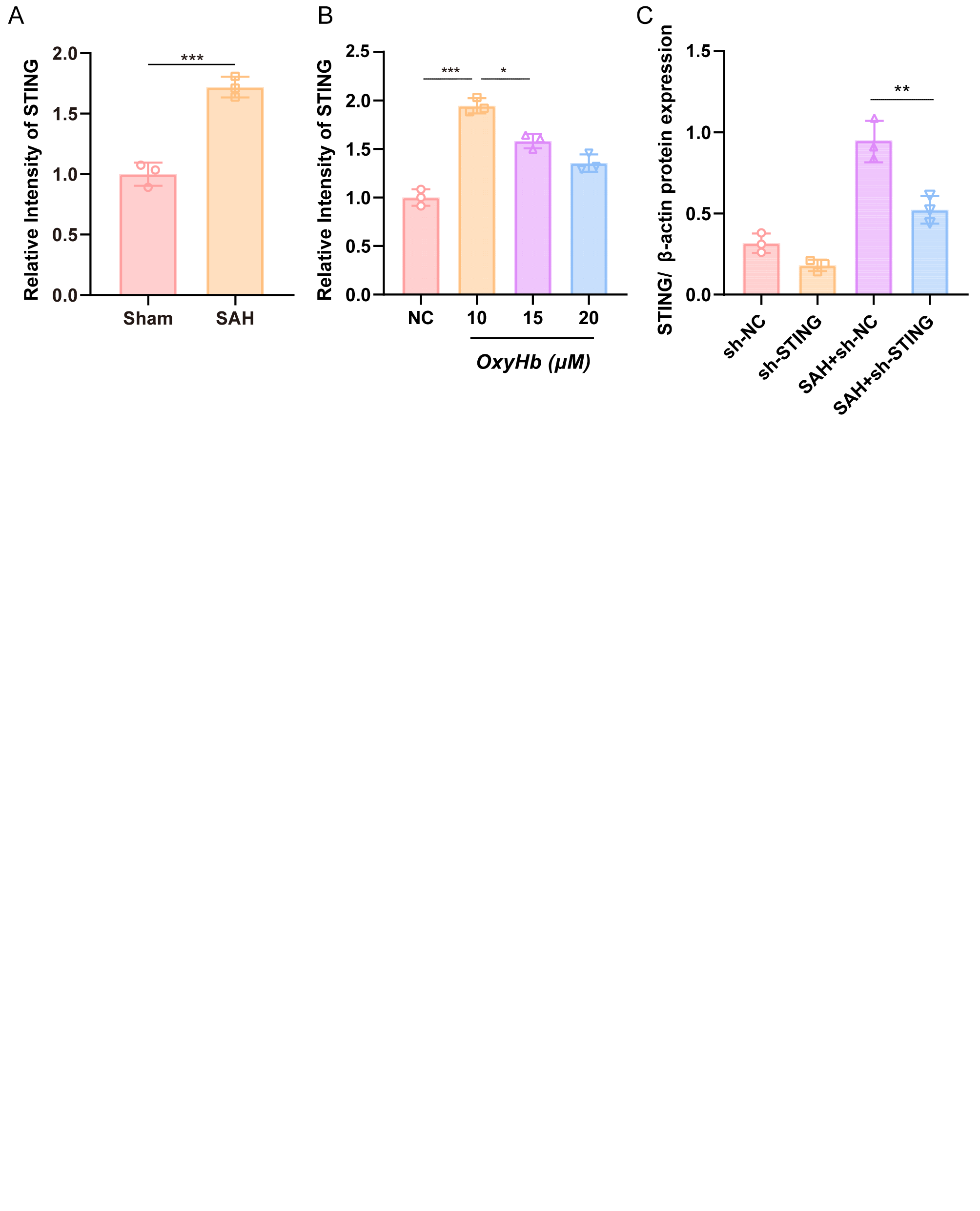
**Figure S1. Semi-quantitative analyses corresponding to Figure 1.** (A) Semi-quantitative analyses corresponding to Figure 1. (B) Semi-quantification of STING fluorescence intensity in BV-2 cells after stimulation with different concentrations of OxyHb. (C) Quantification of STING protein expression normalized to β-actin in the indicated groups. Data are presented as mean ± SEM (n = 3). Statistical analysis was performed using an unpaired Student’s t-test for two-group comparisons and one-way ANOVA followed by Tukey’s multiple-comparison test for multiple-group comparisons. **p* < 0.05, ***p* < 0.01, ****p* < 0.001


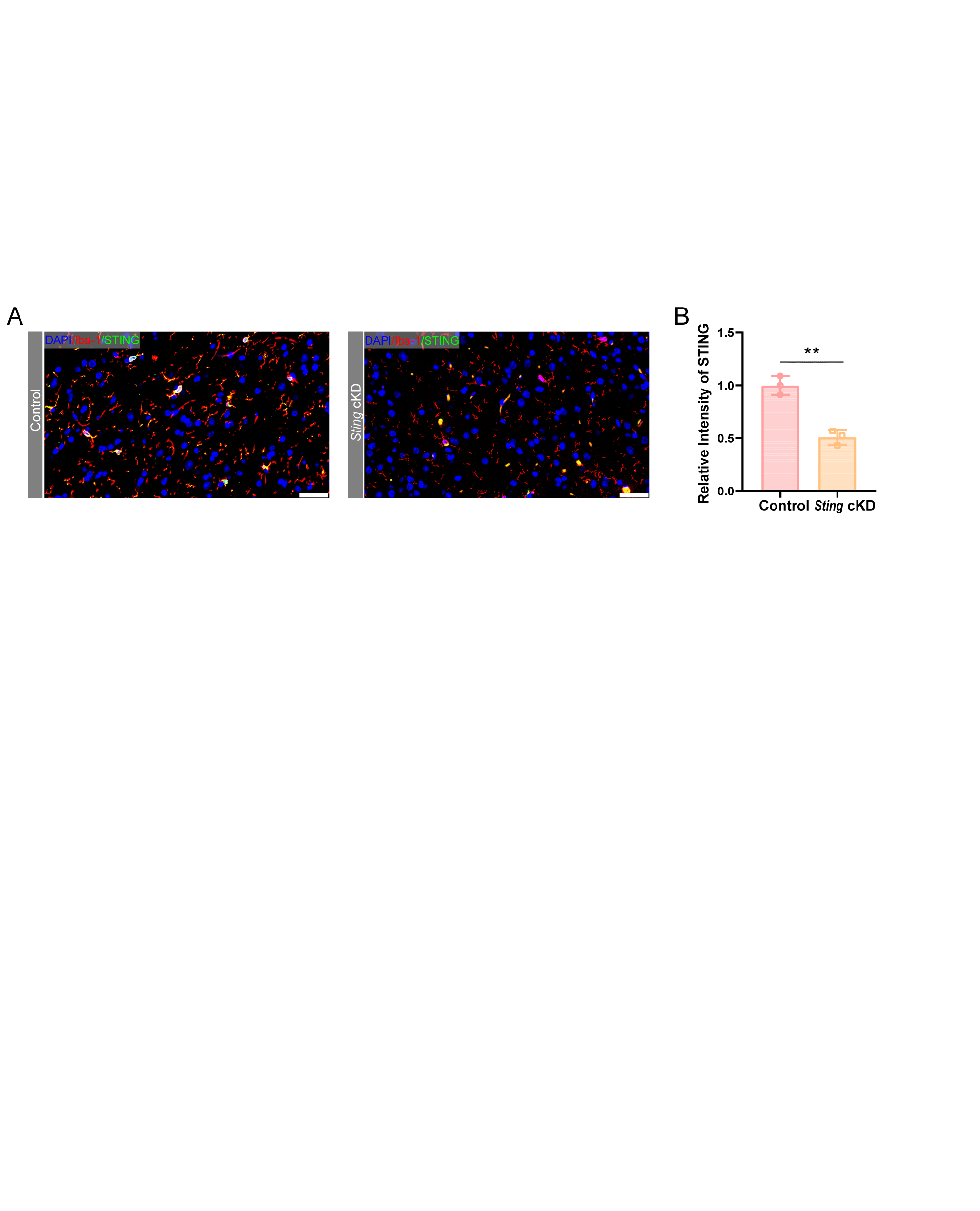


**Figure S2. AAV-mediated conditional knockdown efficiency of STING in microglia.** (A) Representative immunofluorescence images showing STING expression in Iba1-positive microglia from control and Sting cKD mice after AAV delivery. Iba1, red; STING, green; DAPI, blue. Scale bar: 40 μm.(B) Quantification of STING fluorescence intensity in Iba1-positive microglia. Data are presented as mean ± SEM (n = 3). Statistical analysis was performed using an unpaired Student’s t-test. ***p* < 0.01.


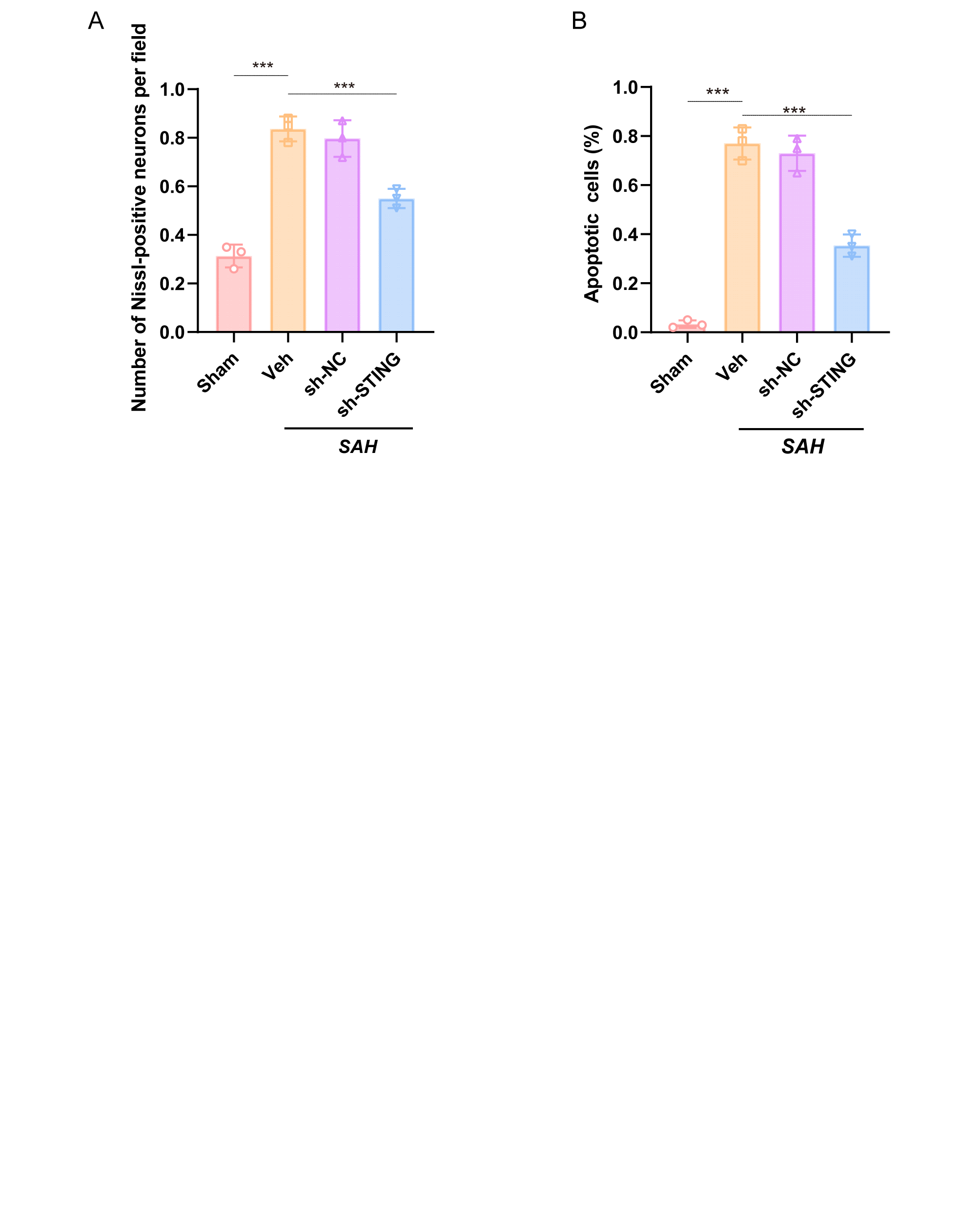


**Figure S3. STING knockdown alleviates neuronal injury after SAH.** (A) Quantification of Nissl-positive neurons per field in the Sham, SAH + Veh, SAH + sh-NC, and SAH + sh-STING groups. (B) Quantification of TUNEL-positive apoptotic cells in the Sham, SAH + Veh, SAH + sh-NC, and SAH + sh-STING groups. Data are presented as mean ± SEM (n = 3). Statistical analysis was performed using one-way ANOVA followed by Tukey’s multiple-comparison test. ****p* < 0.001.


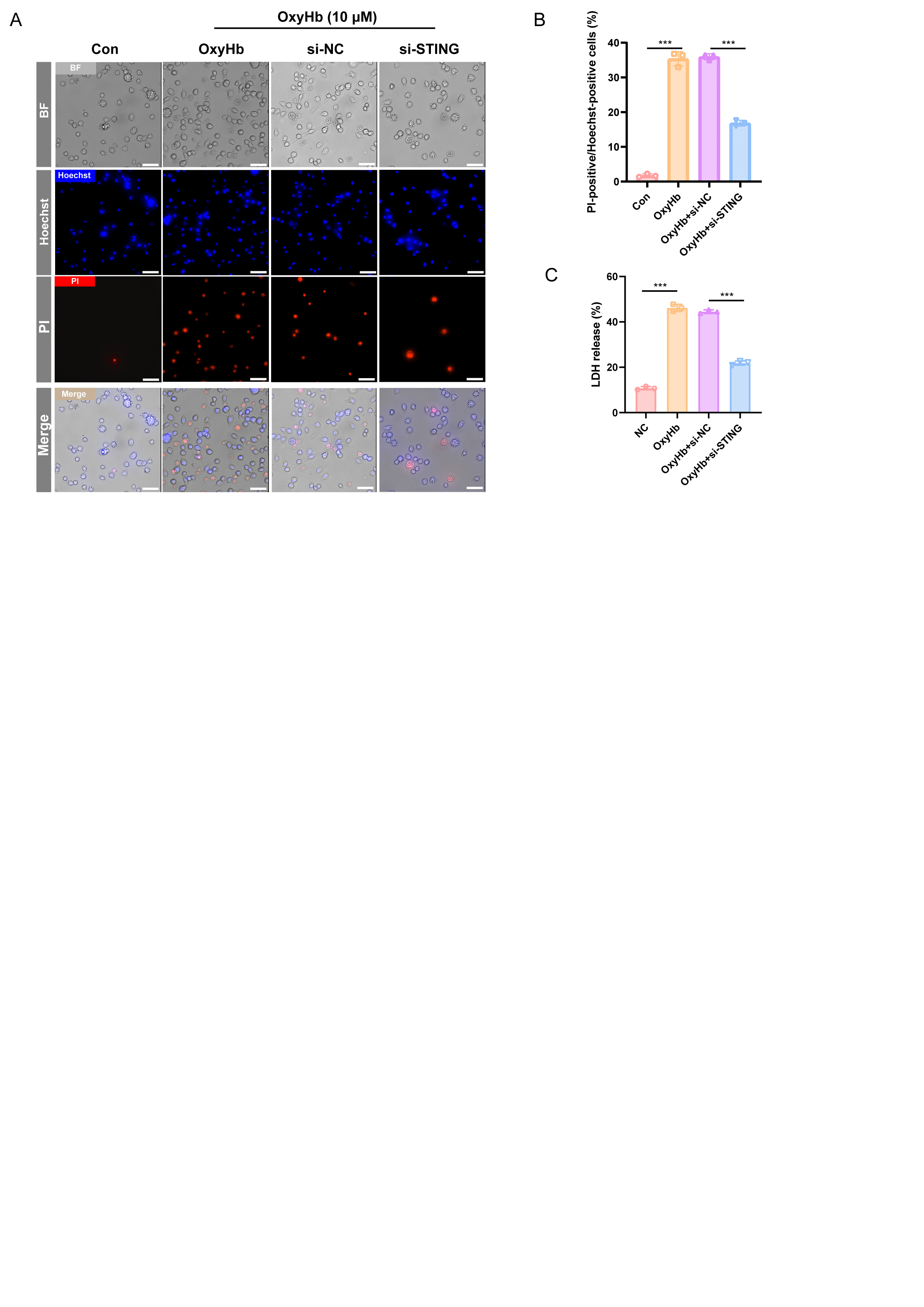


**Figure S4. PI/Hoechst 33342 double staining of BV-2 cells under different treatments.** (A) Representative bright-field and fluorescence images of BV-2 cells stained with Hoechst 33342 and PI. OxyHb stimulation markedly increased PI-positive cells, whereas STING knockdown reduced PI uptake. Hoechst 33342, blue; PI, red. Scale bar: 50 μm. (B) Quantification of PI-positive/Hoechst-positive cells in each group. LDH release assay showing increased membrane damage in OxyHb-treated BV-2 cells and its attenuation by STING knockdown. Data are presented as mean ± SEM (n = 3). Statistical analysis was performed using one-way ANOVA followed by Tukey’s multiple-comparison test. ****p* < 0.001.


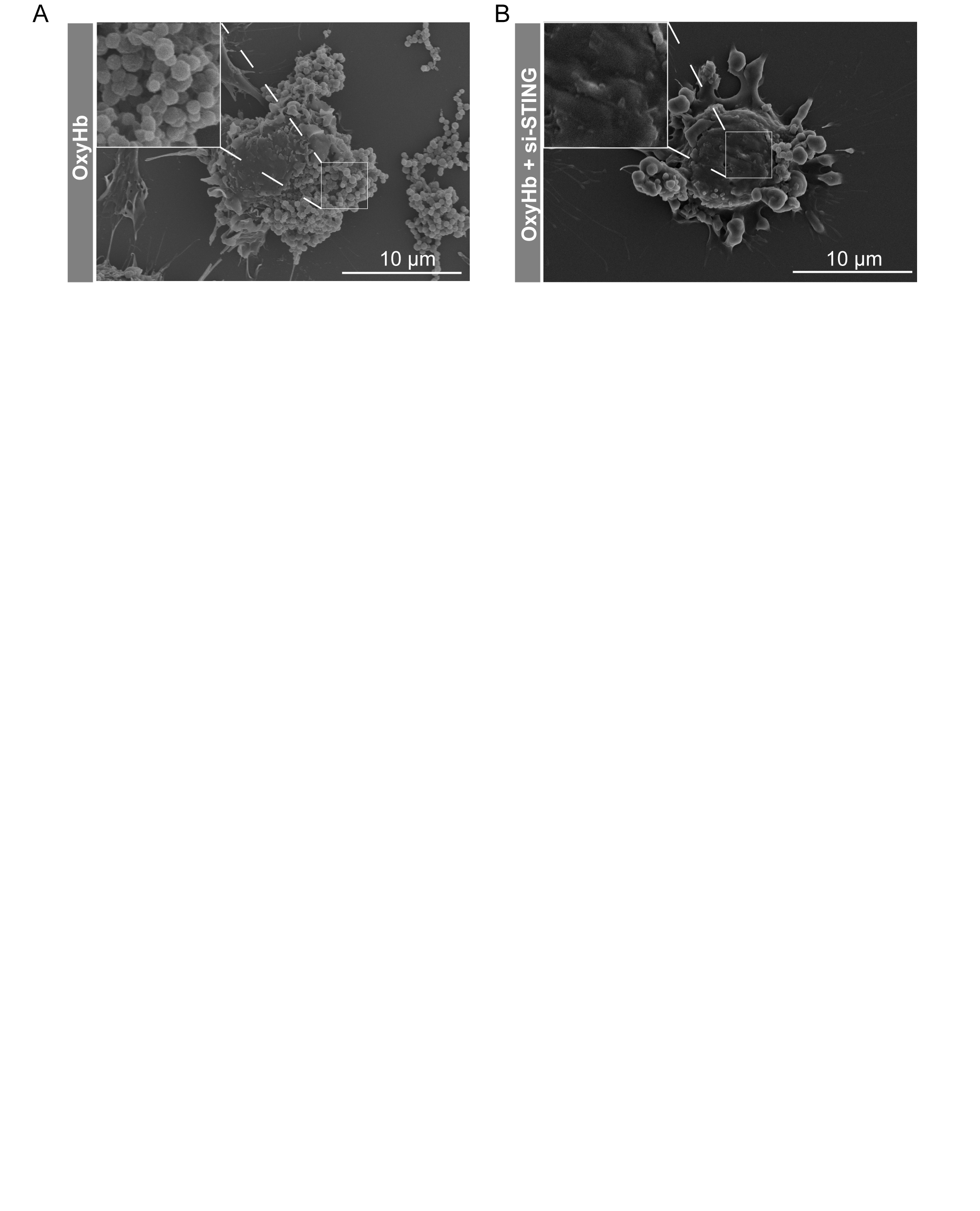


**Figure S5. High-magnification SEM images showing pyroptosis-associated morphological changes in OxyHb-treated BV-2 cells**. (A) Representative high-magnification SEM image of BV-2 cells after OxyHb stimulation, showing typical pyroptosis-associated morphological features, including cell swelling, membrane disruption, membrane ballooning, and abundant pyroptotic body-like structures on the cell surface. The inset shows an enlarged view of the boxed region. (B) Representative high-magnification SEM image of BV-2 cells in the OxyHb + si-STING group, showing attenuated membrane disruption and reduced pyroptotic body-like structures after STING knockdown. The inset shows an enlarged view of the boxed region. Scale bar: 10 μm.


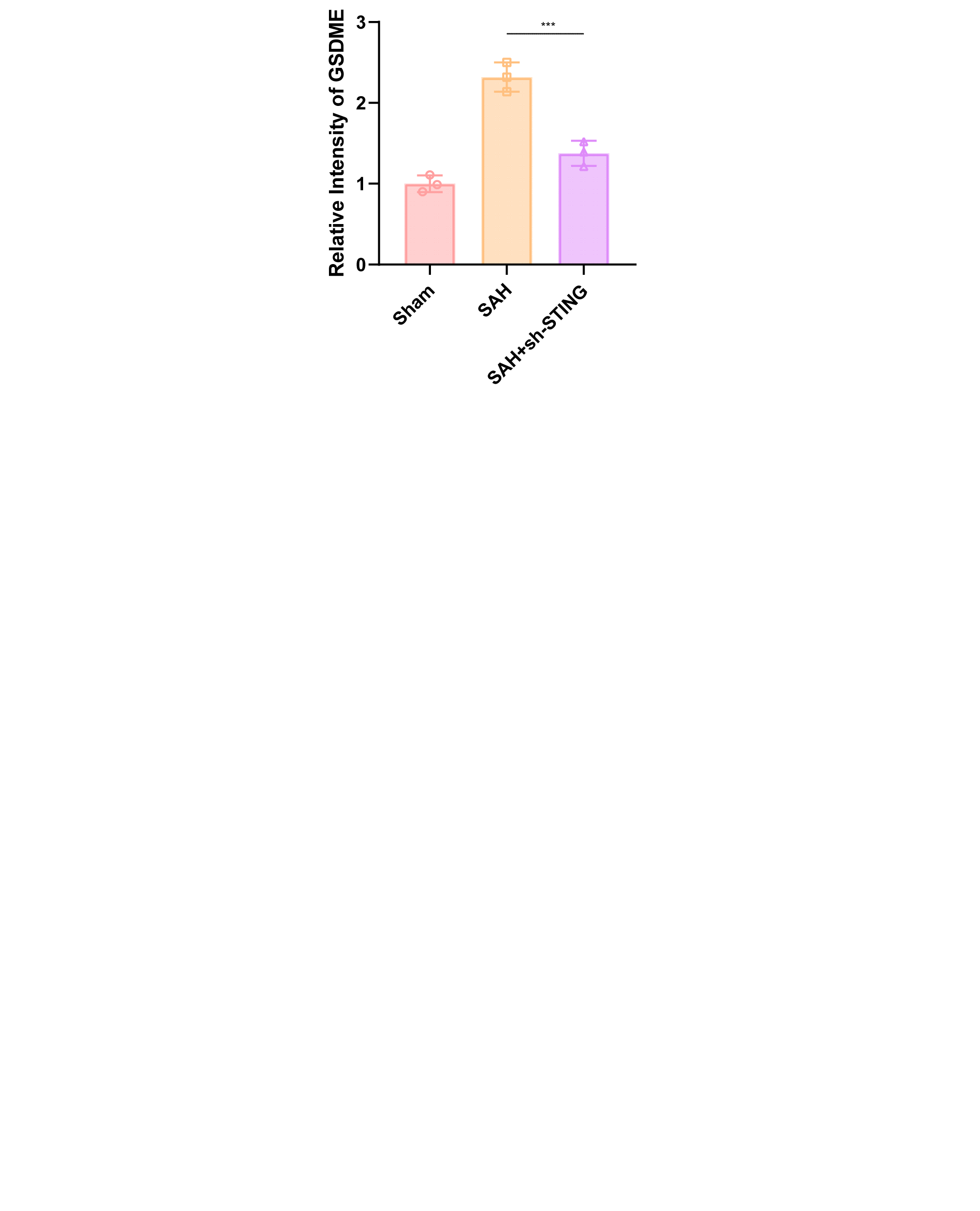
**Figure S6. STING knockdown reduces GSDME expression after SAH.** Semi-quantification of GSDME fluorescence intensity in brain tissues from Sham, SAH, and SAH + sh-STING groups based on immunofluorescence staining. Data are presented as mean ± SEM (n = 3). Statistical analysis was performed using one-way ANOVA followed by Tukey’s multiple-comparison test. ****p* < 0.001.


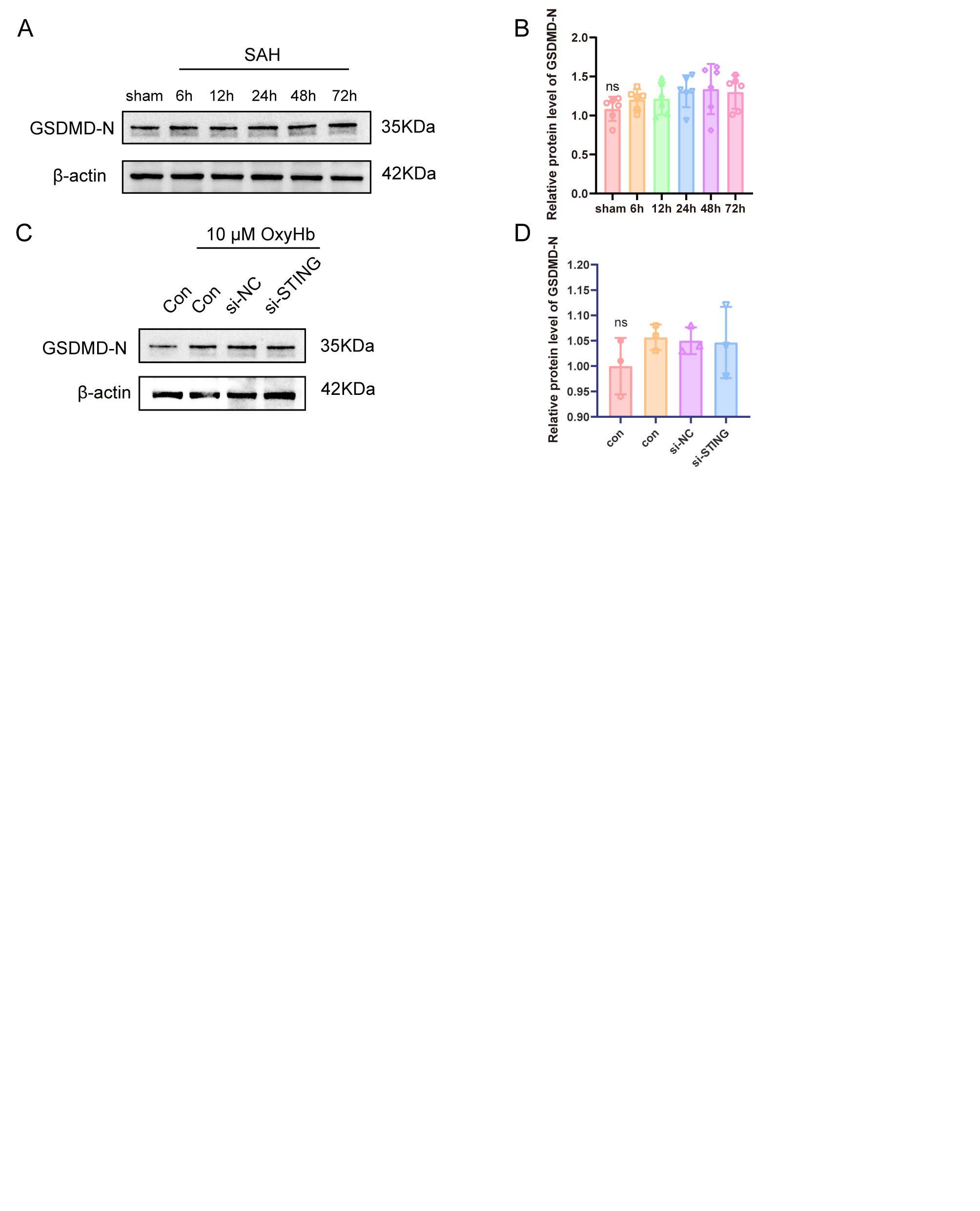


**Figure S7. GSDMD-N shows no prominent alteration in the SAH mouse model or OxyHb-stimulated BV-2 cells**. (A) Western blot analysis of GSDMD-N expression in ipsilateral cortical tissues from Sham mice and SAH mice at 6, 12, 24, 48, and 72 h after SAH. β-actin was used as the loading control. (B) Densitometric quantification of GSDMD-N protein levels in brain tissues from the indicated groups, normalized to β-actin. (C) Western blot analysis of GSDMD-N expression in BV-2 cells under the indicated treatments. BV-2 cells were exposed to OxyHb (10 μM), with or without si-NC or si-STING transfection. β-actin was used as the loading control. (D) Densitometric quantification of GSDMD-N protein levels in BV-2 cells from the indicated groups, normalized to β-actin. Data are presented as mean ± SEM (n = 3). Statistical analysis was performed using one-way ANOVA followed by Tukey’s multiple-comparison test. ns, not significant.


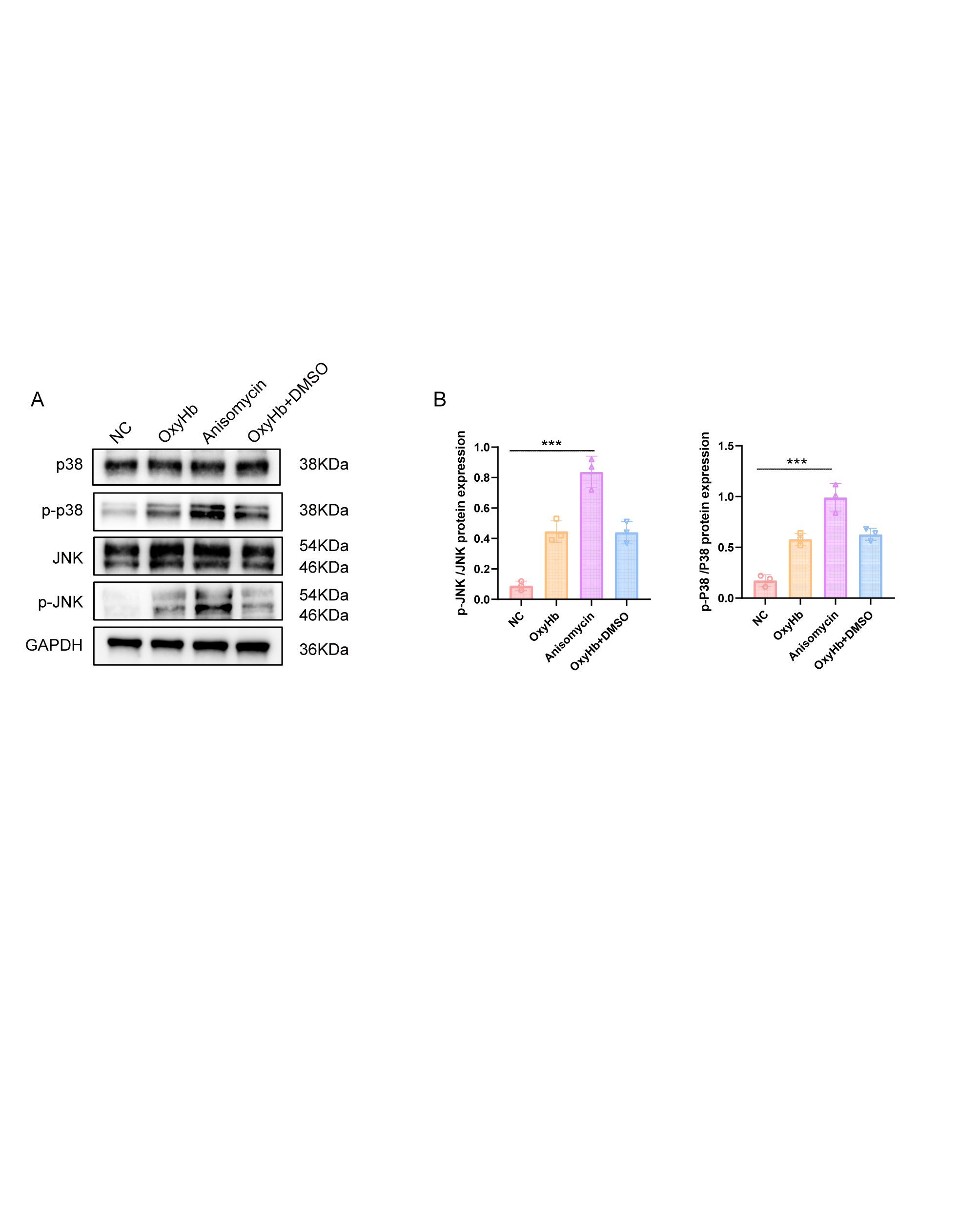
**Figure S8. Anisomycin activates MAPK signaling in microglial cells.** (A) Representative western blot images showing the expression of p38, phosphorylated p38 (p-p38), JNK, and phosphorylated JNK (p-JNK) in microglial cells treated with NC, OxyHb, anisomycin, or Oxy-Hb + DMSO. GAPDH was used as the loading control. (B) Quantification of p-JNK/JNK and p-p38/p38 protein expression levels in different groups. Data are presented as mean ± SEM (n = 3). Statistical analysis was performed using one-way ANOVA followed by Tukey’s multiple-comparison test. ****p* < 0.001.


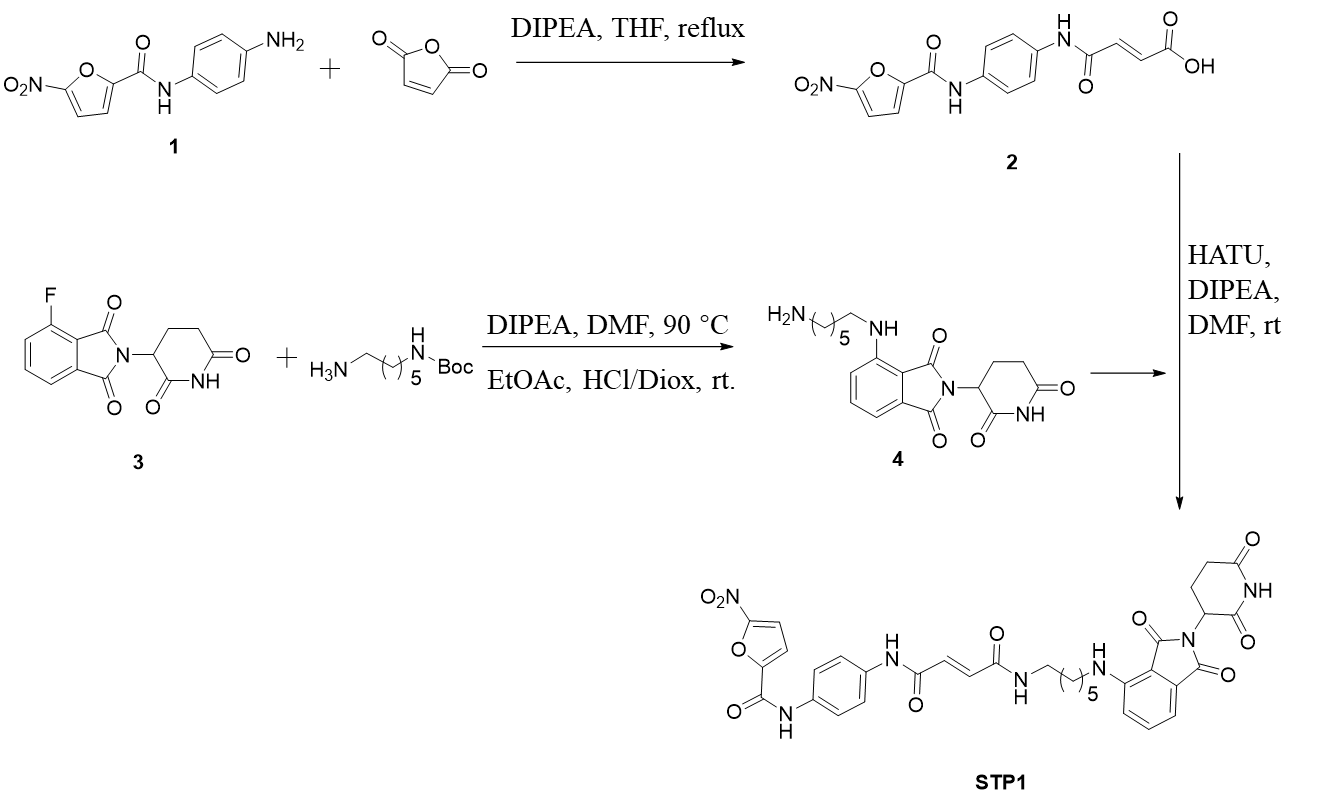
**Figure S9. Synthetic route of STP1.** Schematic representation of the synthetic procedure for STP1, including the preparation of intermediates (1–4) and final amide coupling to obtain the target molecule.


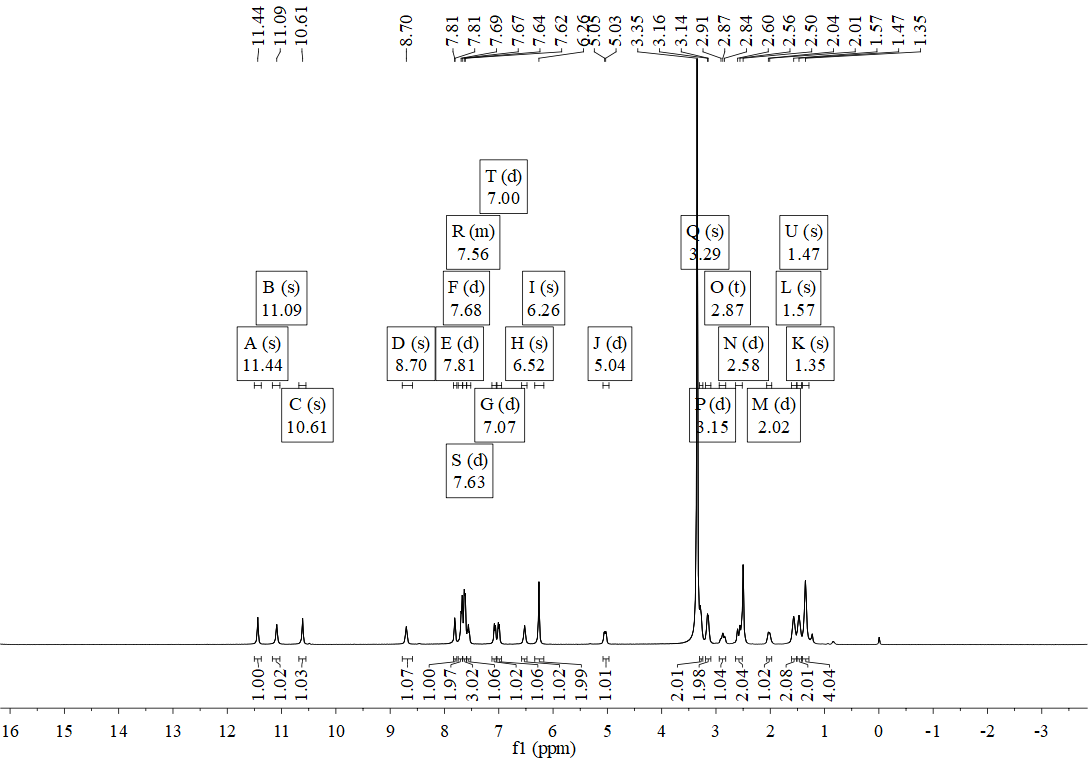


**Figure S10. ^1^HNMR Spectrum of Compound STP1. ^1^**HNMR spectrum of STP1 recorded in DMSO-d6 at 400 MHz, showing characteristic resonances at δ 11.44, 11.09, and 10.61 ppm for NH protons; δ 8.70–6.52 ppm for aromatic and heteroaromatic protons; δ 6.26 ppm for olefinic protons; δ 5.04 ppm for the methine proton; and δ 3.29–1.35 ppm for aliphatic linker protons.


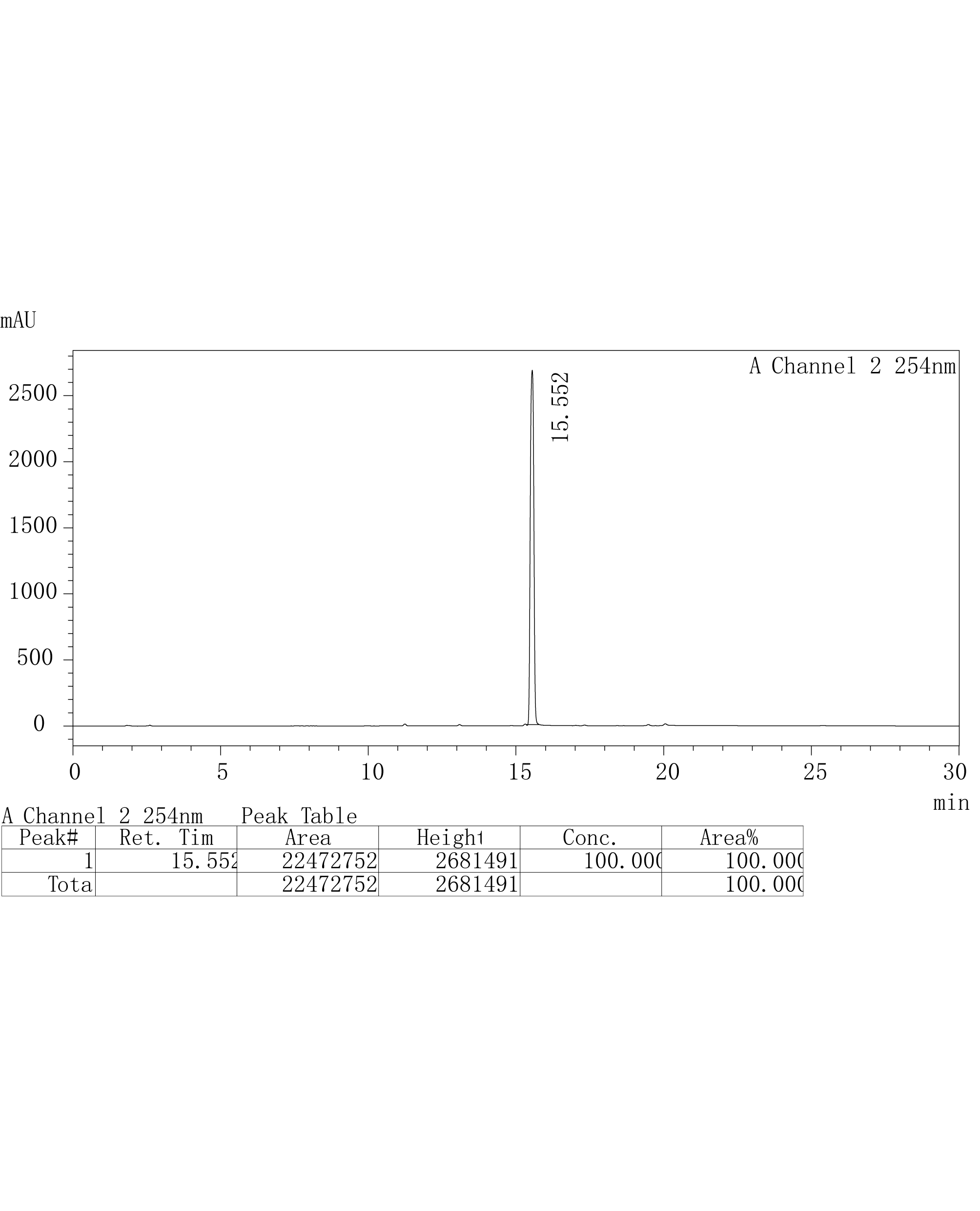
**Figure S11. HPLC chromatogram of STP1.** HPLC analysis of STP1 was performed using a Shimadzu LC-20AT system equipped with an ODS column. The mobile phase was methanol/water containing 0.5‰ TFA at a flow rate of 1.0 mL/min. The purity of STP1 was determined to be 100.000%.


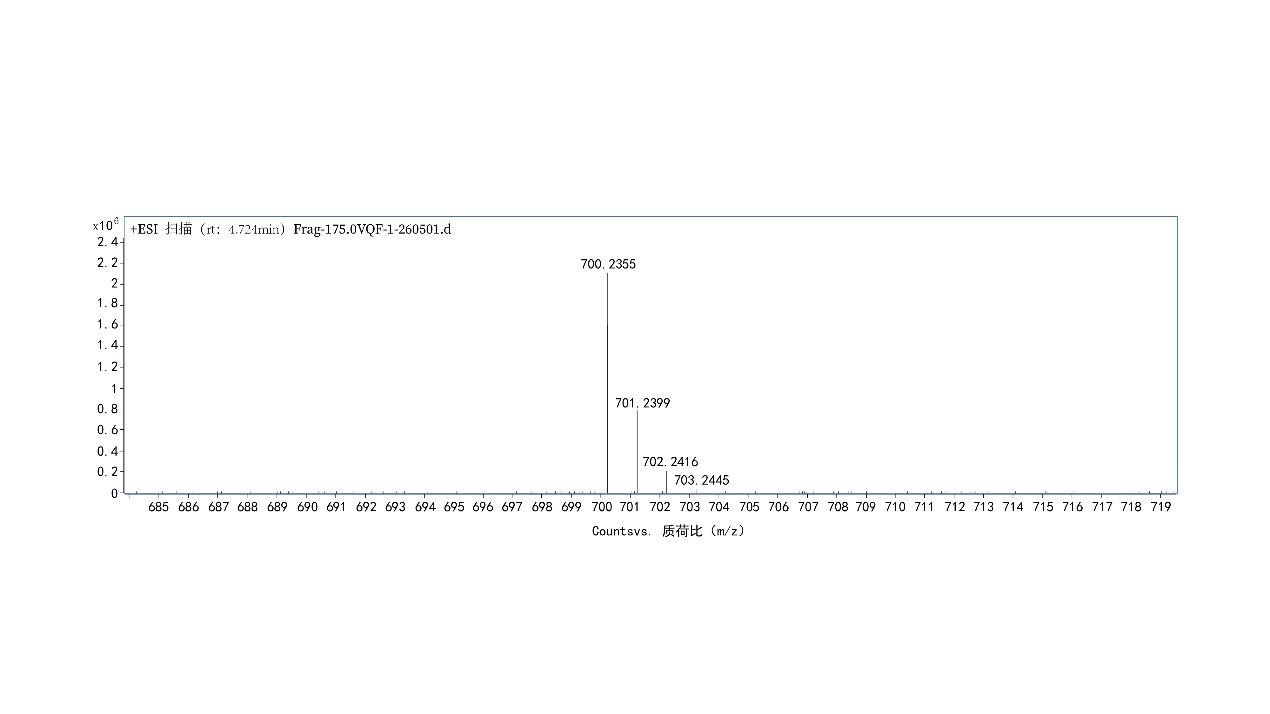
**Figure S12. HRMS Spectra of compound STP1 from LC-MS.** LC-MS/HRMS analysis showed the expected molecular ion peak of STP1 at m/z 700.2353, consistent with the calculated value of 700.2367 for [M+H] ^+.


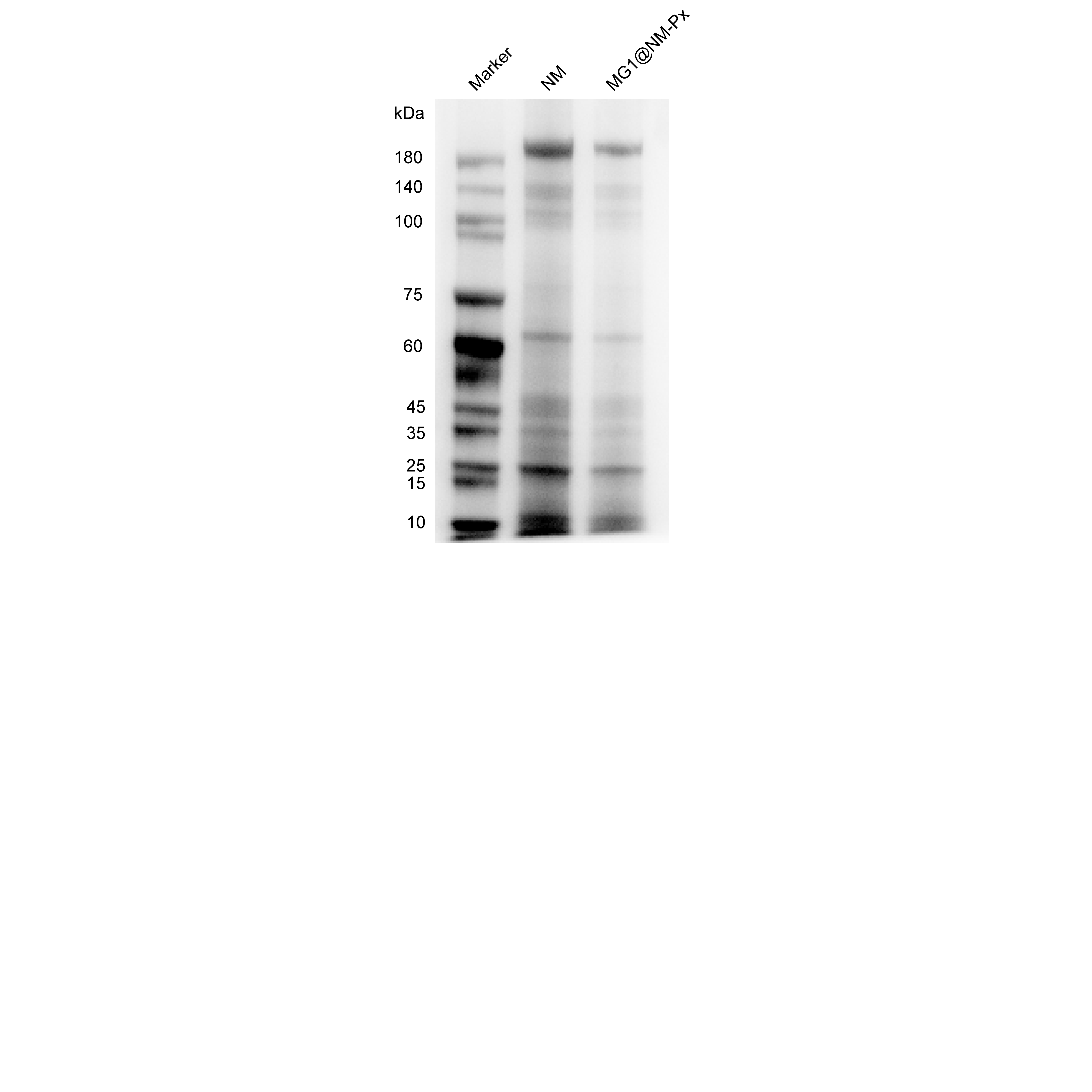
**Figure S13. Coomassie brilliant blue staining/SDS-PAGE analysis of neutrophil membrane protein retention.** Protein-band profiles of neutrophil membrane (NM) and MG1@NM-Px were analyzed by SDS-PAGE followed by Coomassie brilliant blue staining. MG1@NM-Px retained multiple protein bands with a distribution pattern broadly consistent with that of the NM fraction, supporting successful neutrophil membrane coating and preservation of membrane-associated proteins.


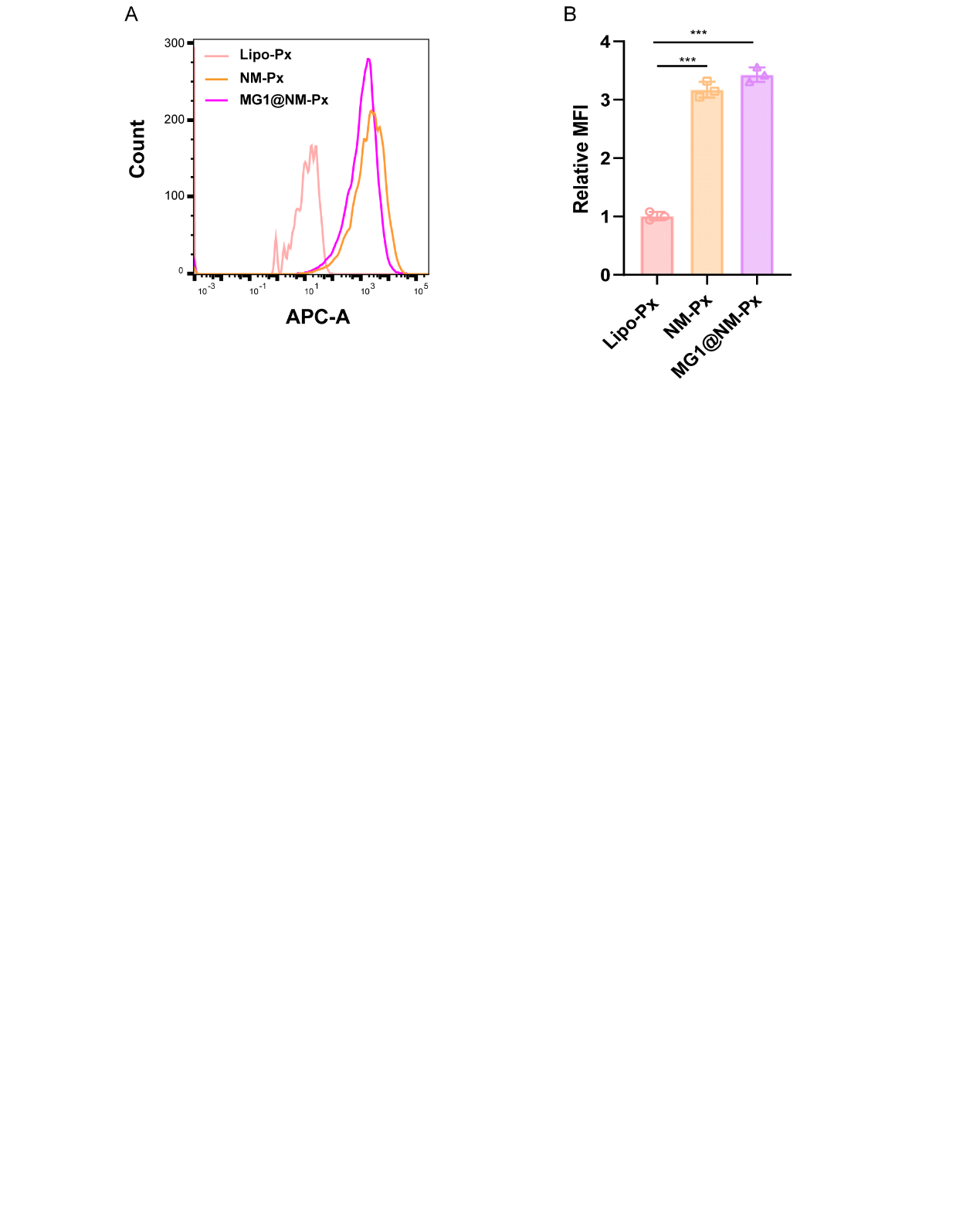
**Figure S14. Flow cytometry-based validation of neutrophil membrane-mediated inflammatory endothelial association.** (A) Representative flow cytometry histograms showing cell-associated DiD fluorescence in TNF-α-activated endothelial cells after incubation with DiD-labeled Lipo-Px, NM-Px, or MG1@NM-Px. (B) Quantitative analysis of relative mean fluorescence intensity (MFI). MFI values were normalized to the Lipo-Px group. NM-Px and MG1@NM-Px showed significantly increased endothelial association compared with uncoated Lipo-Px, indicating preserved neutrophil membrane-associated endothelial interaction function. Data are presented as mean ± SEM (n = 3). ****p* < 0.001.


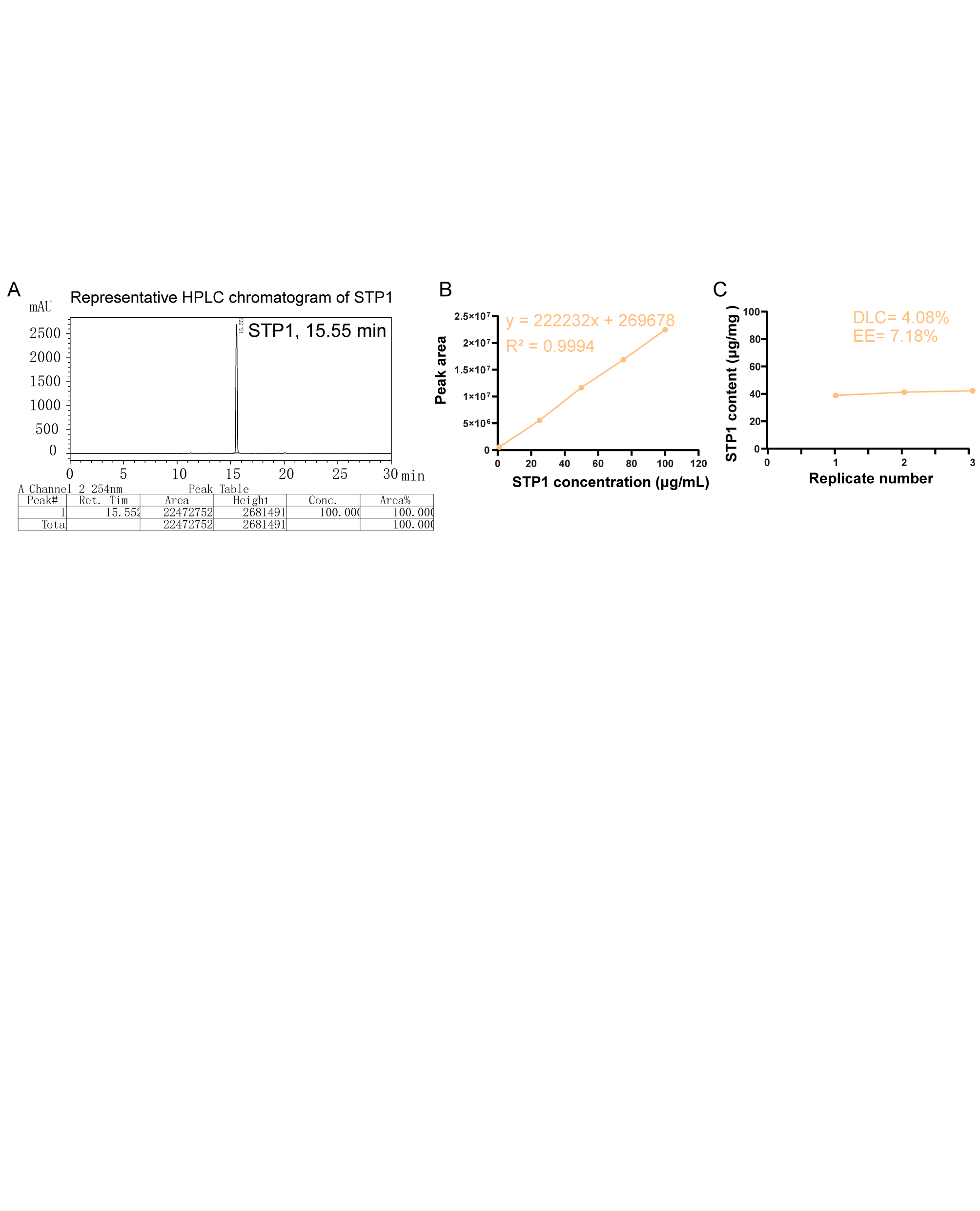
**Figure S15. HPLC-based quantification of STP1 loading in the liposomal formulation.** (A) Representative HPLC chromatogram of STP1 showing a characteristic peak at approximately 15.55 min. (B) Calibration curve of STP1 determined by HPLC. The linear regression equation was y = 222232x + 269678, with R² = 0.9994. (C) Quantification of STP1 content in the liposomal formulation based on HPLC analysis. The average STP1 content was 40.8 μg/mg nanoparticles, corresponding to a drug loading content (DLC) of 4.08 wt% and an encapsulation efficiency (EE) of 7.18%. Data are presented as mean ± SEM (n = 3).


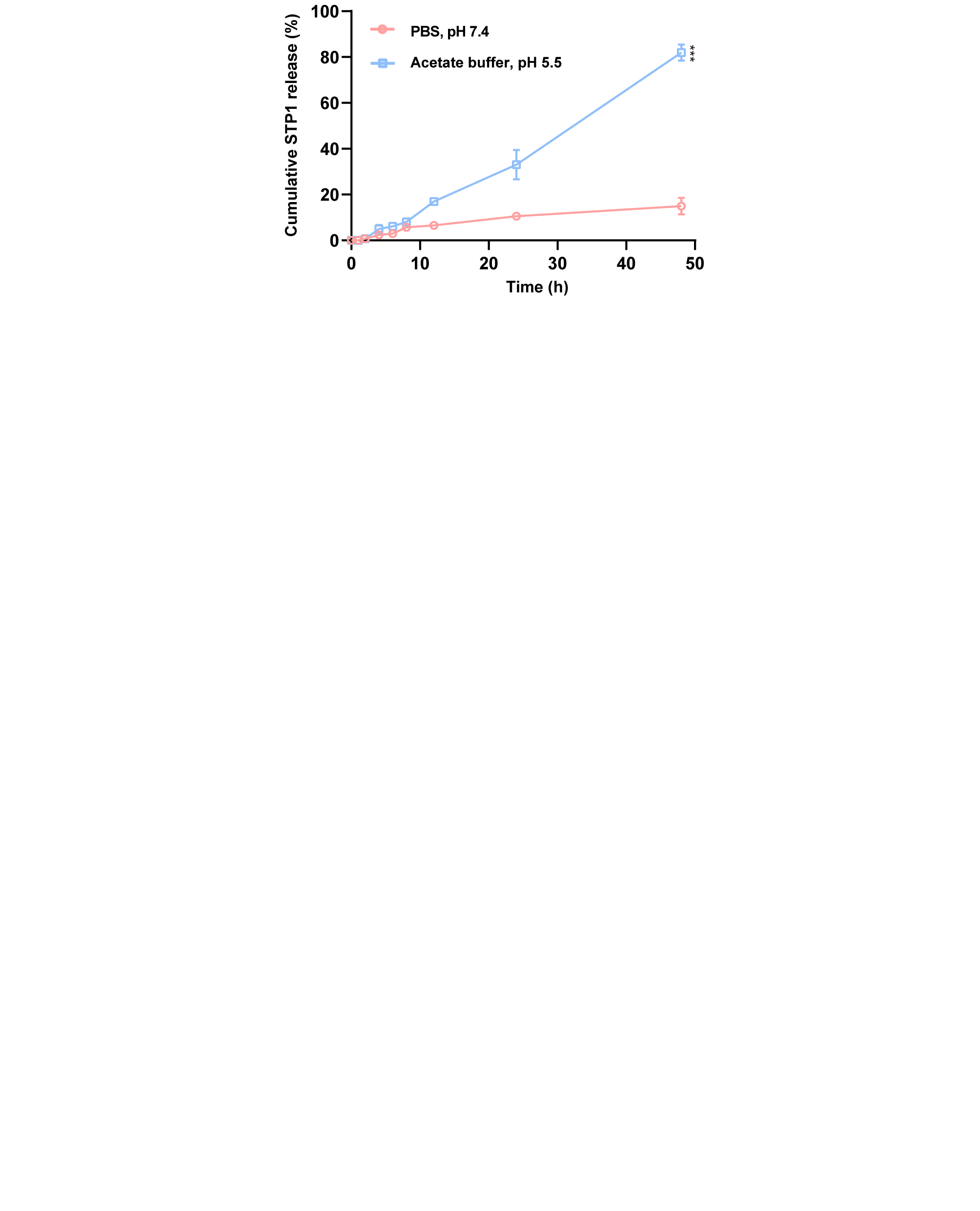
**Figure S16. In vitro release profile of STP1 from MG1@NM-Px under different pH conditions.** Cumulative release of STP1 from MG1@NM-Px was evaluated in PBS at pH 7.4 and acetate buffer at pH 5.5 over 48 h. Data are presented as mean ± SEM (n = 3 independent measurements). Statistical significance was determined by two-way ANOVA followed by Sidak’s multiple-comparison test. ****p* < 0.001.


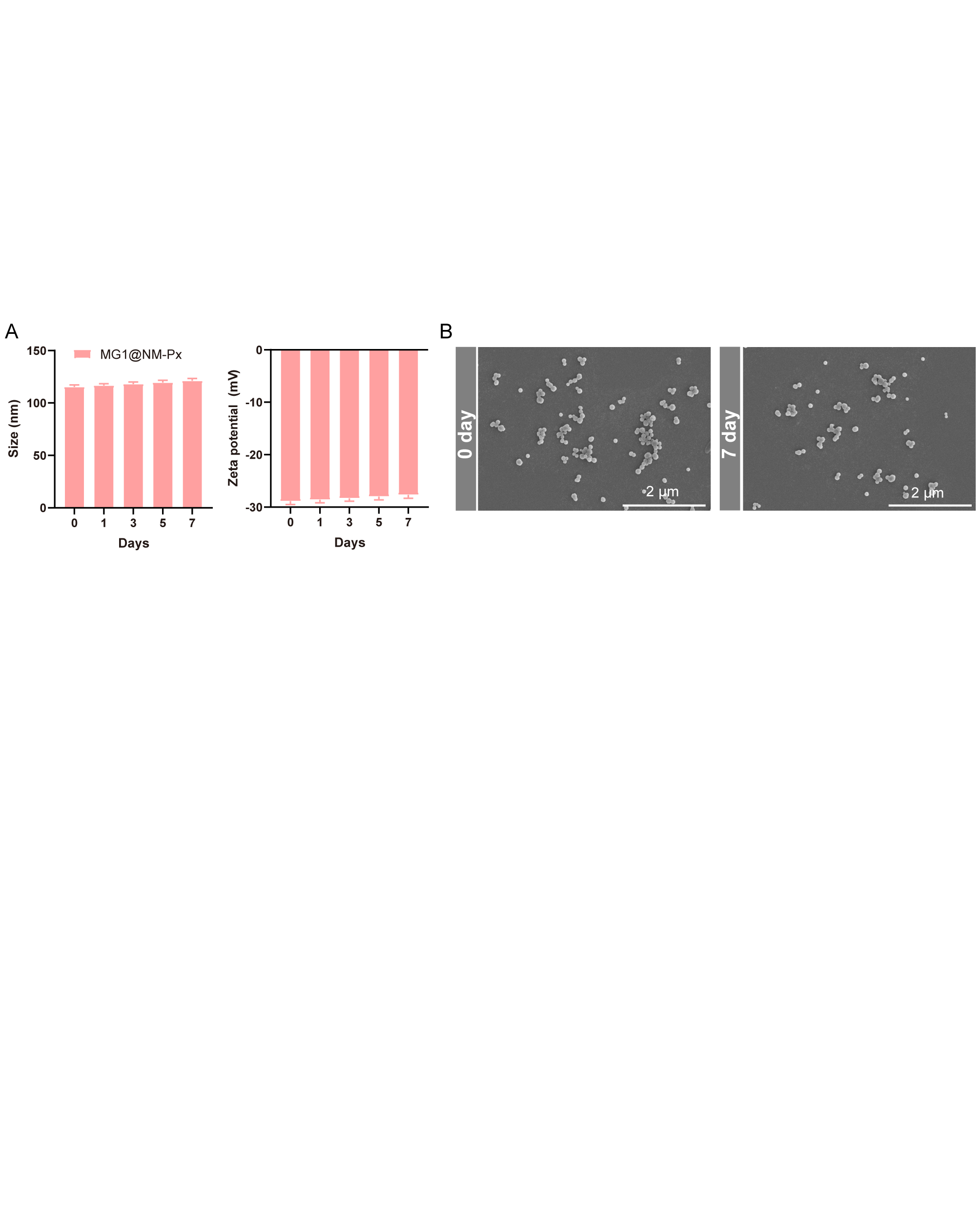
**Figure S17. In vitro stability of MG1@NM-Px under physiological conditions.** (A) Hydrodynamic diameter and zeta potential of MG1@NM-Px during incubation in PBS containing 10% FBS at 37 °C for 7 days. (B) Representative SEM images of MG1@NM-Px after incubation for 0 and 7 days, showing preserved particle morphology without obvious aggregation. Scale bar: 2.0 μm. Data are presented as mean ± SEM (n = 3).


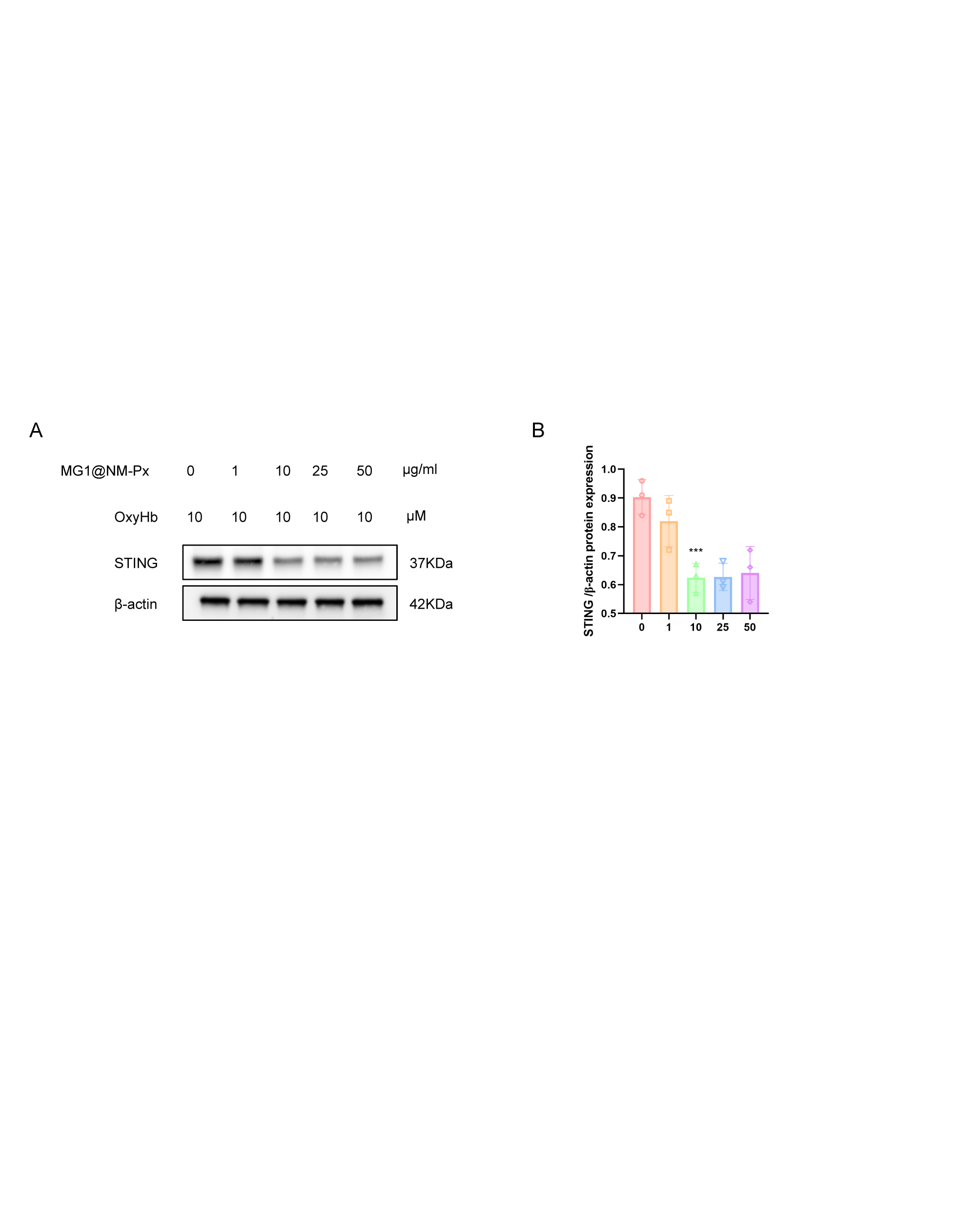


**Figure S18. Determination of the effective working concentration of MG1@NM-Px.** (A) Representative western blot images showing STING protein expression in microglial cells treated with OxyHb (10 μM) and increasing concentrations of MG1@NM-Px (0, 1, 10, 25, and 50 μg mL⁻¹). β-actin was used as the loading control. (B) Quantification of STING/β-actin protein expression levels under different treatment conditions. Data are presented as mean ± SEM (n = 3). ****p* < 0.001.


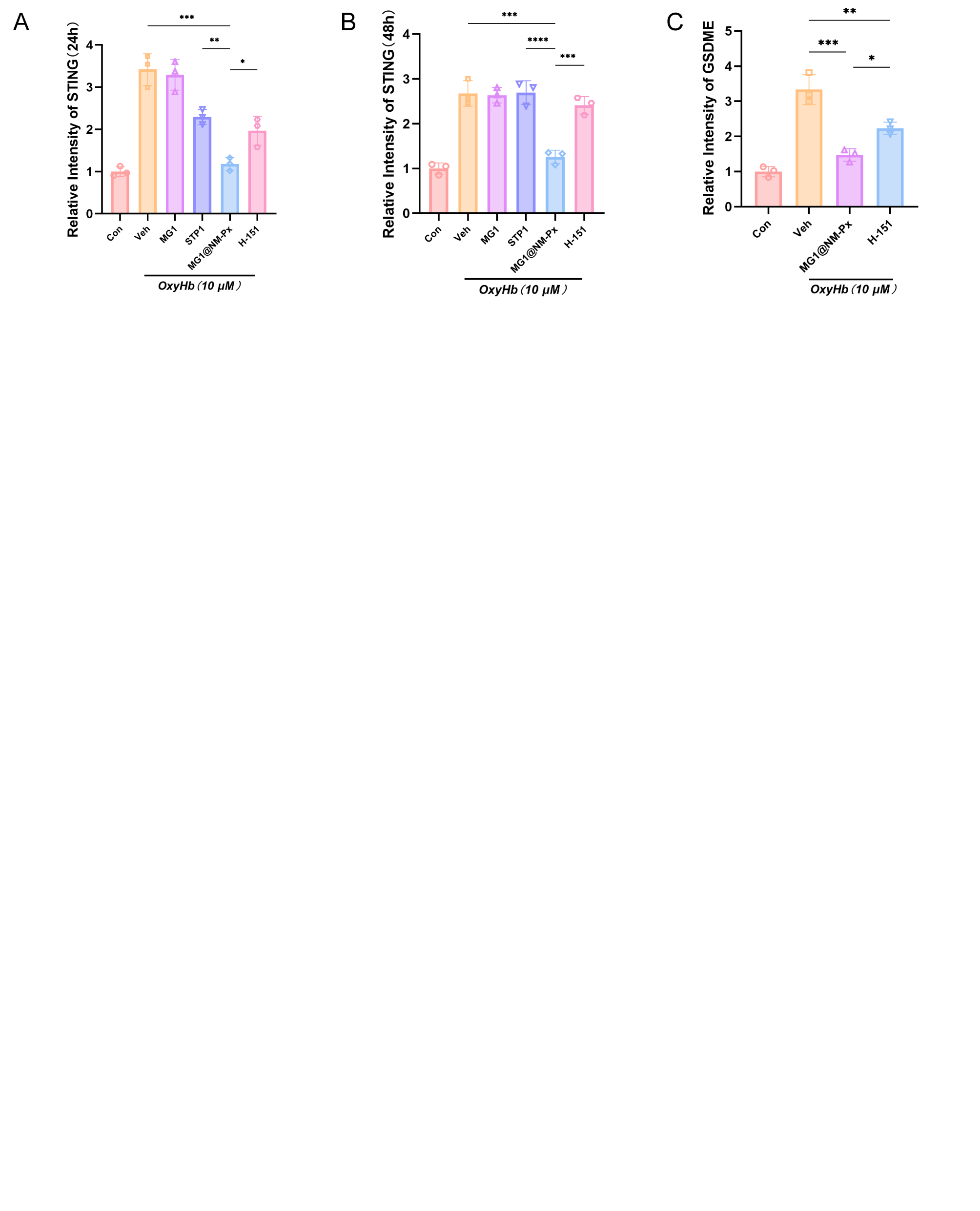
**Figure S19. Effects of different treatments on STING and GSDME expression in OxyHb-stimulated microglial cells.** (A) Semi-quantification of STING fluorescence intensity after 24 h treatment under different conditions. (B) Semi-quantification of STING fluorescence intensity after 48 h treatment under different conditions. (C) Semi-quantification of GSDME fluorescence intensity in OxyHb-stimulated cells after different treatments. Data are presented as mean ± SEM (n = 3). **p* < 0.05, ***p* < 0.01, ****p* < 0.001, *****p* < 0.0001.


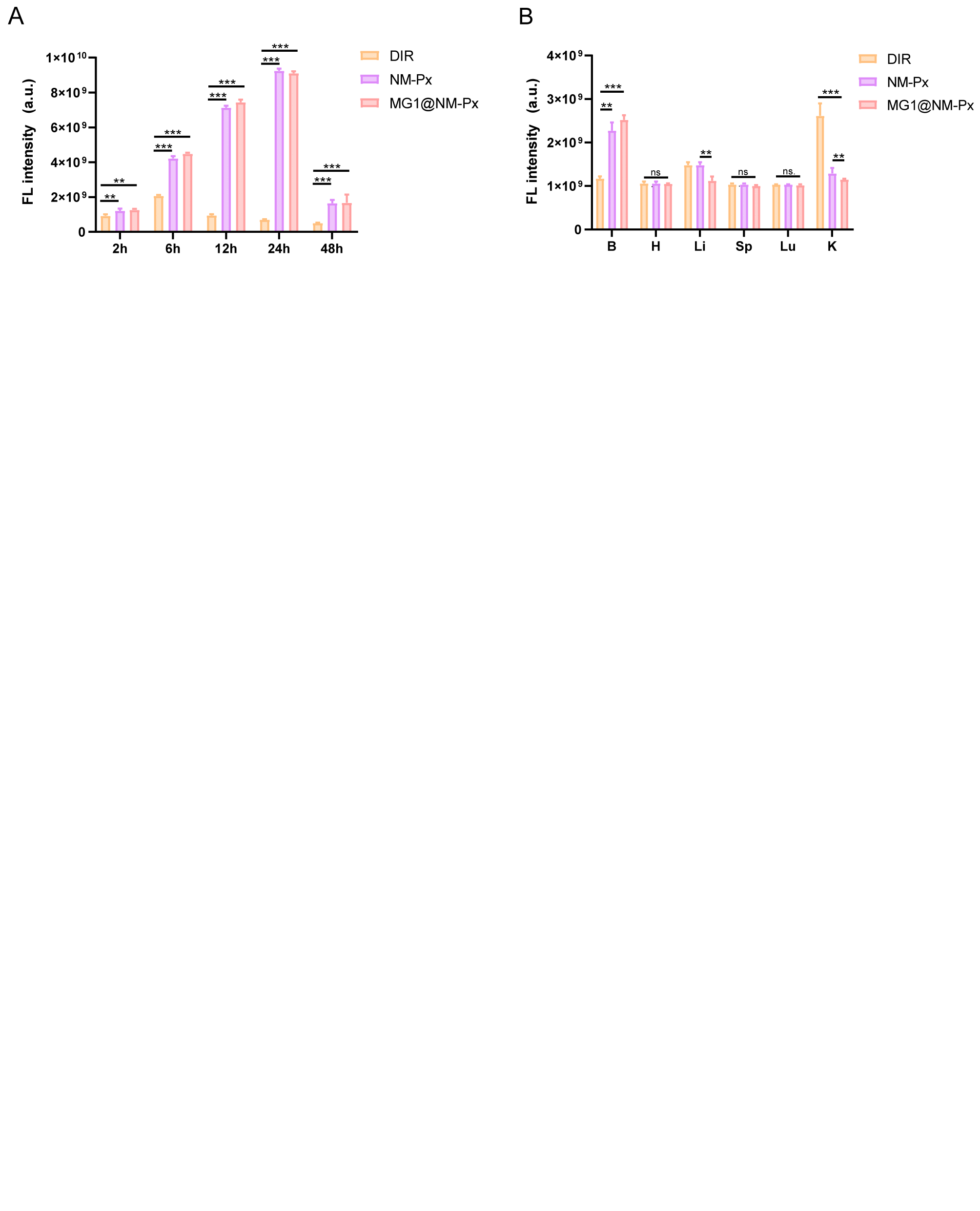
**Figure S20. In vivo fluorescence imaging analysis of nanoparticle biodistribution.** (A) Quantification of fluorescence intensity in brain tissues at different time points (2, 6, 12, 24 and 48 h) after intravenous administration of DiR, NM-Px, or MG1@NM-Px. (B) Quantification of fluorescence intensity in major organs, including B (brain), H (heart), Li (liver), Sp (spleen), Lu (lung), and K (kidney), collected after treatment. Data are presented as mean ± SEM (n = 3). ***p* < 0.01, ****p* < 0.001, ns, not significant.


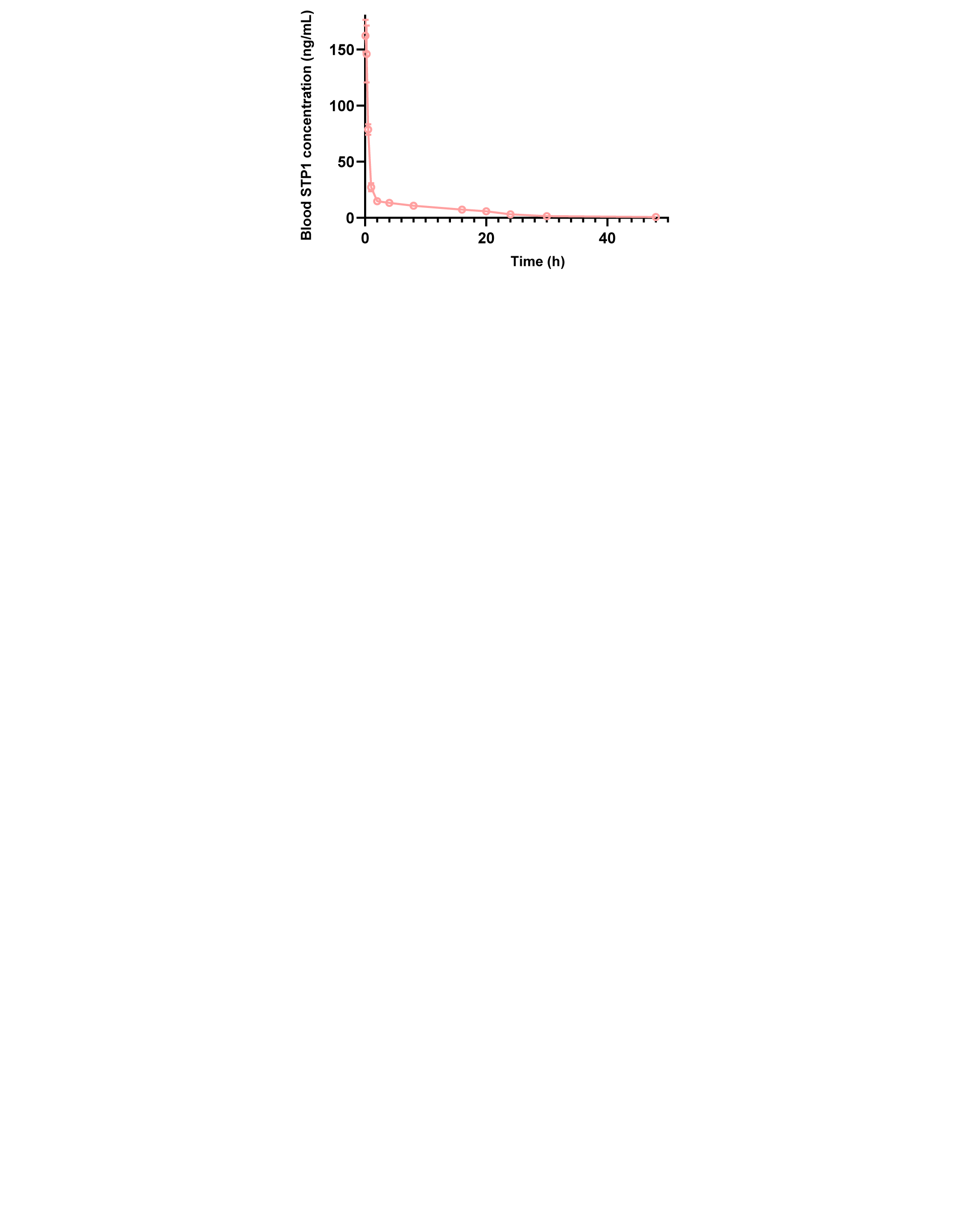
**Figure S21. Blood concentration–time profile of STP1 after intravenous administration of MG1@NM-Px.** MG1@NM-Px was intravenously administered to mice at an STP1-equivalent dose of 8.6 μg per 20 g mouse, corresponding to 0.43 mg/kg. Blood samples were collected at 0.083, 0.25, 0.5, 1, 2, 4, 8, 16, 20, 24, 30, and 48 h after administration, and STP1 concentrations were quantified by HPLC. Data are presented as mean ± SEM (n = 3 mice).


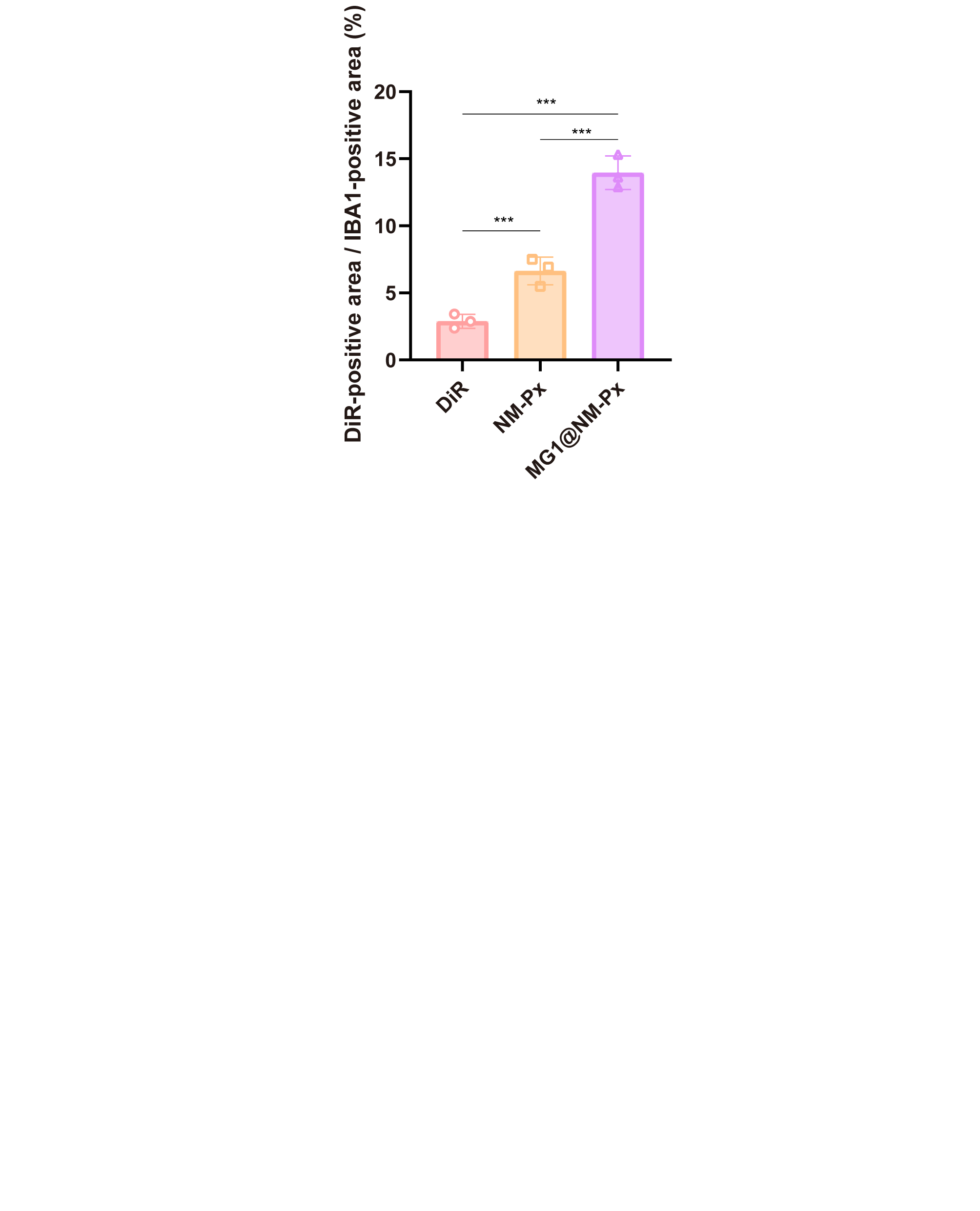
**Figure S22. Semi-quantitative analysis of microglia-associated nanoparticle localization in brain tissues.** Semi-quantification of DiR-positive area within Iba1-positive microglial regions in brain sections from SAH mice after intravenous administration of DiR, NM-Px, or MG1@NM-Px. The ratio of DiR-positive area to Iba1-positive area was used to evaluate microglia-associated nanoparticle localization. Data are presented as mean ± SEM (n = 3). Statistical analysis was performed using one-way ANOVA followed by Tukey‘s multiple-comparison test. ****p* < 0.001.


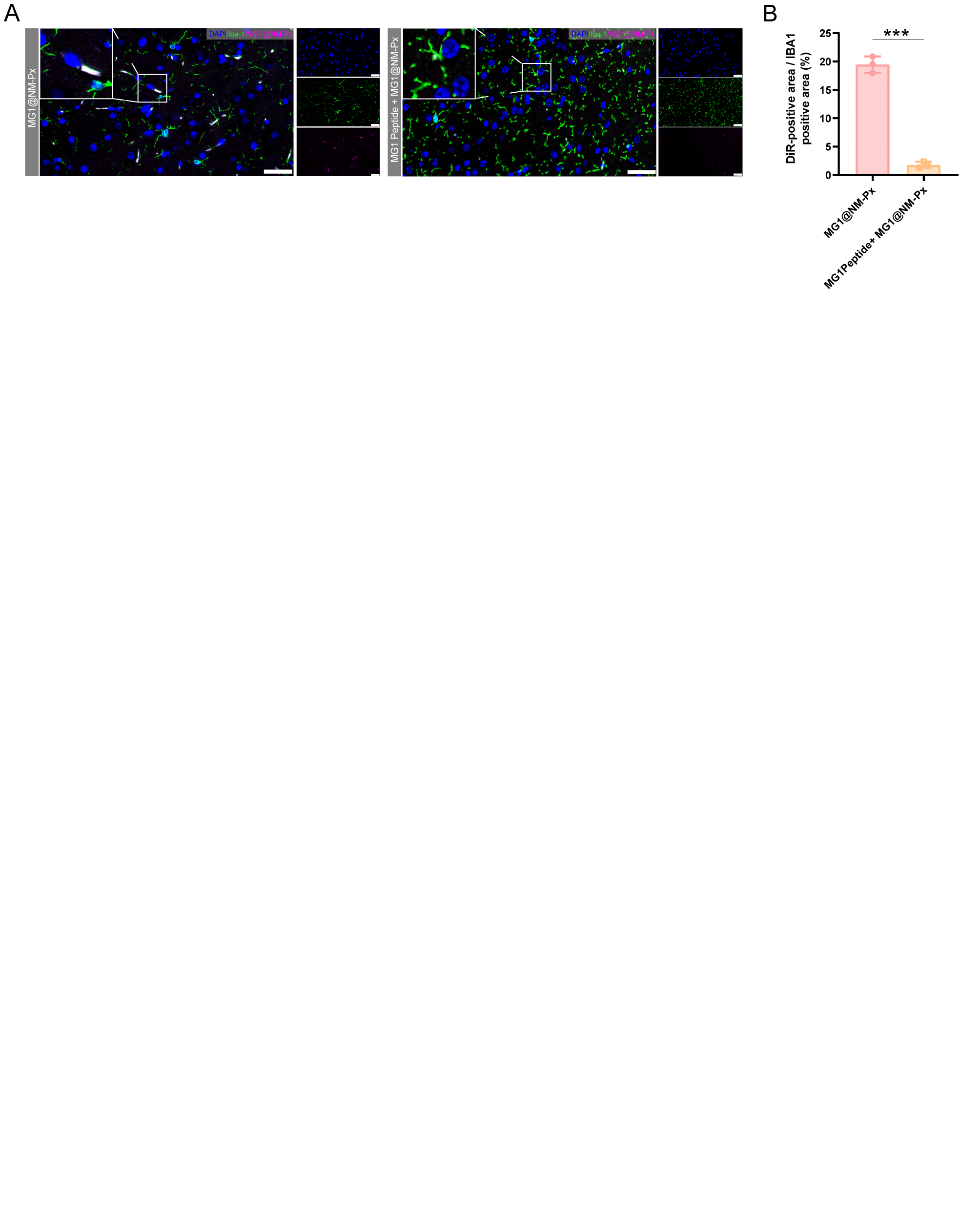
**Figure S23. In vivo competitive blocking experiment for MG1-mediated microglial localization.** (A) Representative immunofluorescence images showing the colocalization of DiR-labeled MG1@NM-Px with Iba1-positive microglia in brain sections from the MG1@NM-Px group and the free MG1 + MG1@NM-Px group. Iba1, green; DiR, red; DAPI, blue. Scale bar: 40 μm. (B) Semi-quantitative analysis of the colocalization between DiR fluorescence and Iba1-positive microglia. Data are presented as mean ± SEM (n = 3). ****p* < 0.001.


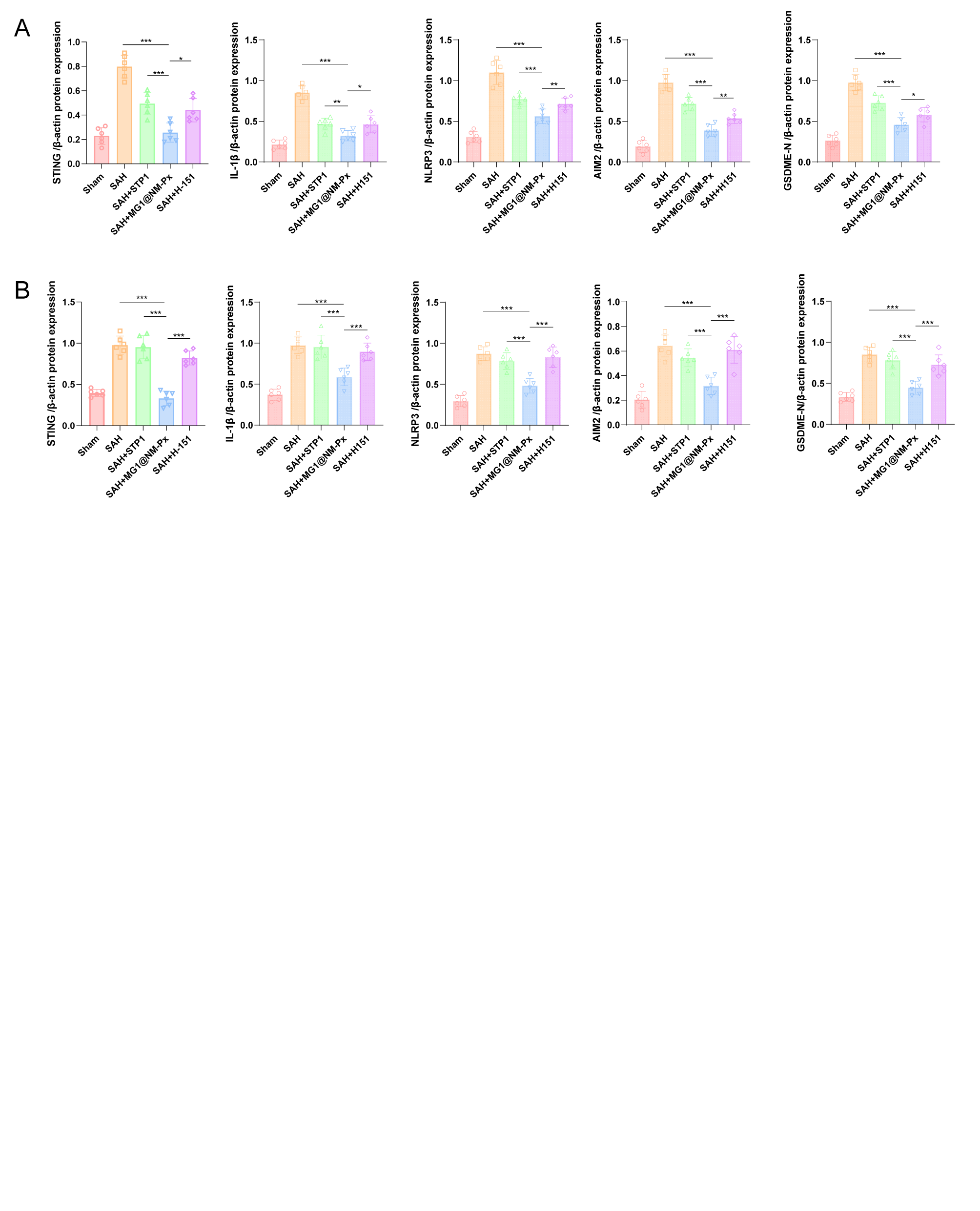
**Figure S24. Quantitative analysis of immunoblot results shown in Figure 6D.** (A) Densitometric quantification of STING and pyroptosis-related proteins, including IL-1β, NLRP3, AIM2, and cleaved GSDME-N, in brain tissues collected 24 h after treatment. (B) Densitometric quantification of STING and pyroptosis-related proteins in brain tissues collected 48 h after treatment. Protein expression levels were normalized to β-actin. Data are presented as mean ± SEM (n = 6). **p* < 0.05, ***p* < 0.01, ****p* < 0.001.


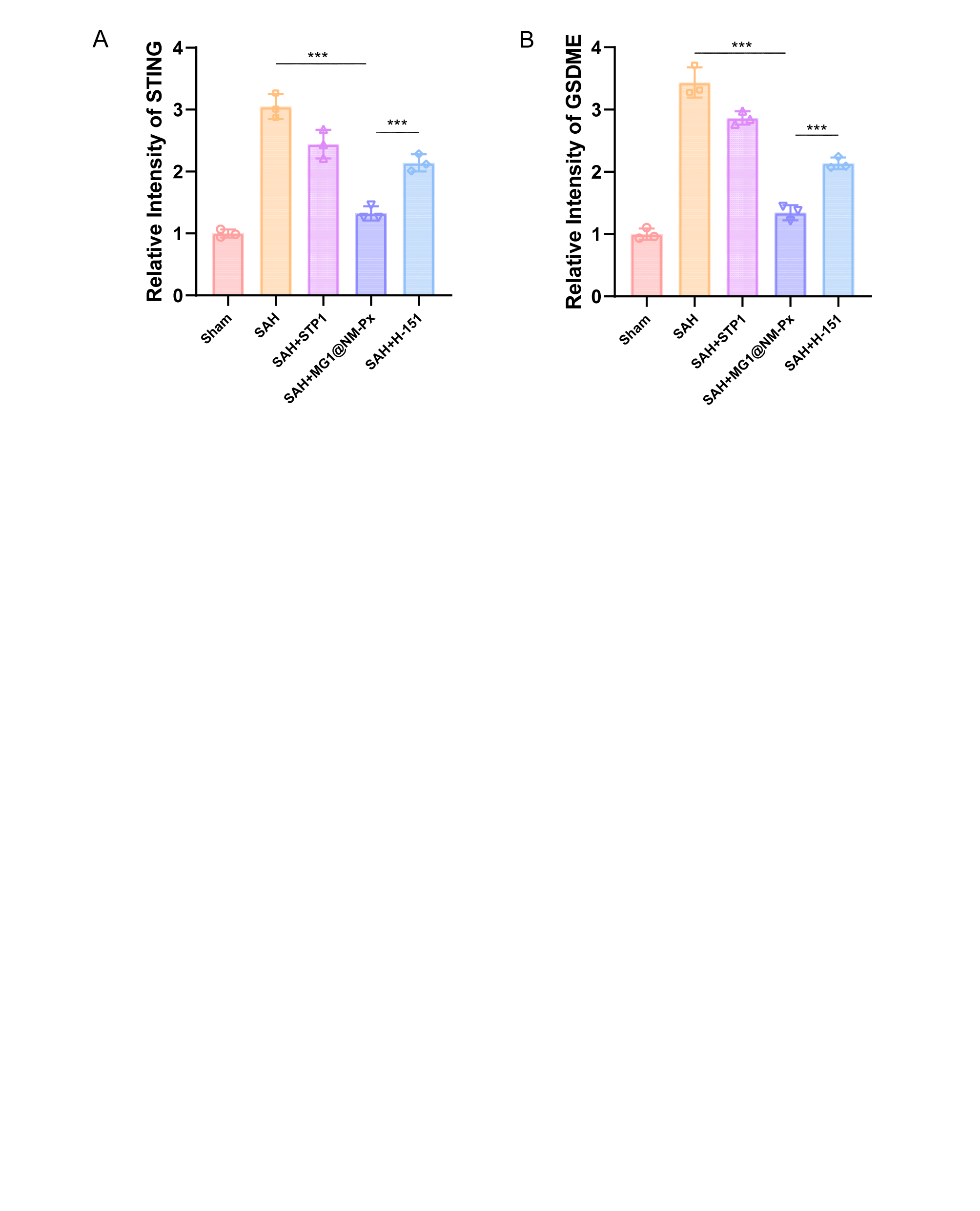
**Figure S25. Semi-quantitative analysis of STING degradation and GSDME-associated pyroptotic signaling in brain tissues after treatment.** (A) Semi-quantification of STING fluorescence intensity in brain sections from Sham, SAH, SAH + STP1, SAH + MG1@NM-Px, and SAH + H-151 mice. MG1@NM-Px markedly reduced STING fluorescence intensity compared with the SAH + STP1 and SAH + H-151 groups. (B) Semi-quantification of GSDME fluorescence intensity in brain sections from the indicated groups. MG1@NM-Px substantially decreased GSDME fluorescence intensity, supporting its inhibitory effect on GSDME-associated pyroptotic signaling after SAH. Data are presented as mean ± SEM (n = 3). Statistical analysis was performed using one-way ANOVA followed by Tukey’s multiple-comparison test. ****p* < 0.001.


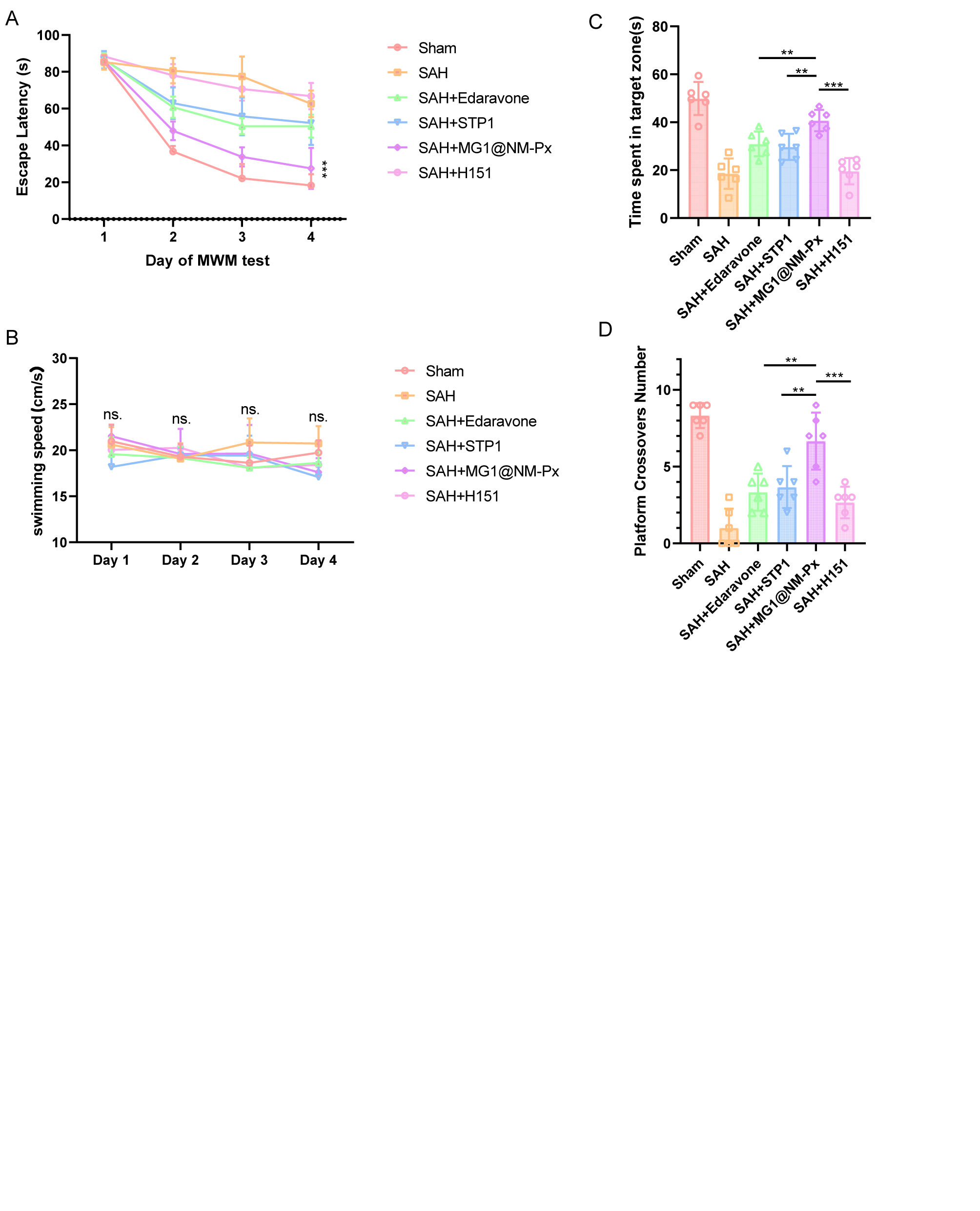
**Figure S26. Morris water maze test evaluating cognitive function after SAH.** (A) Escape latency during the four consecutive days of training in the Morris water maze test. (B) Swimming speed during the training period. (C) Time spent in the target quadrant during the probe trial. (D) Number of platform crossings during the probe trial. Data are presented as mean ± SEM (n = 6). ***p* < 0.01, ****p* < 0.001; ns, not significant.


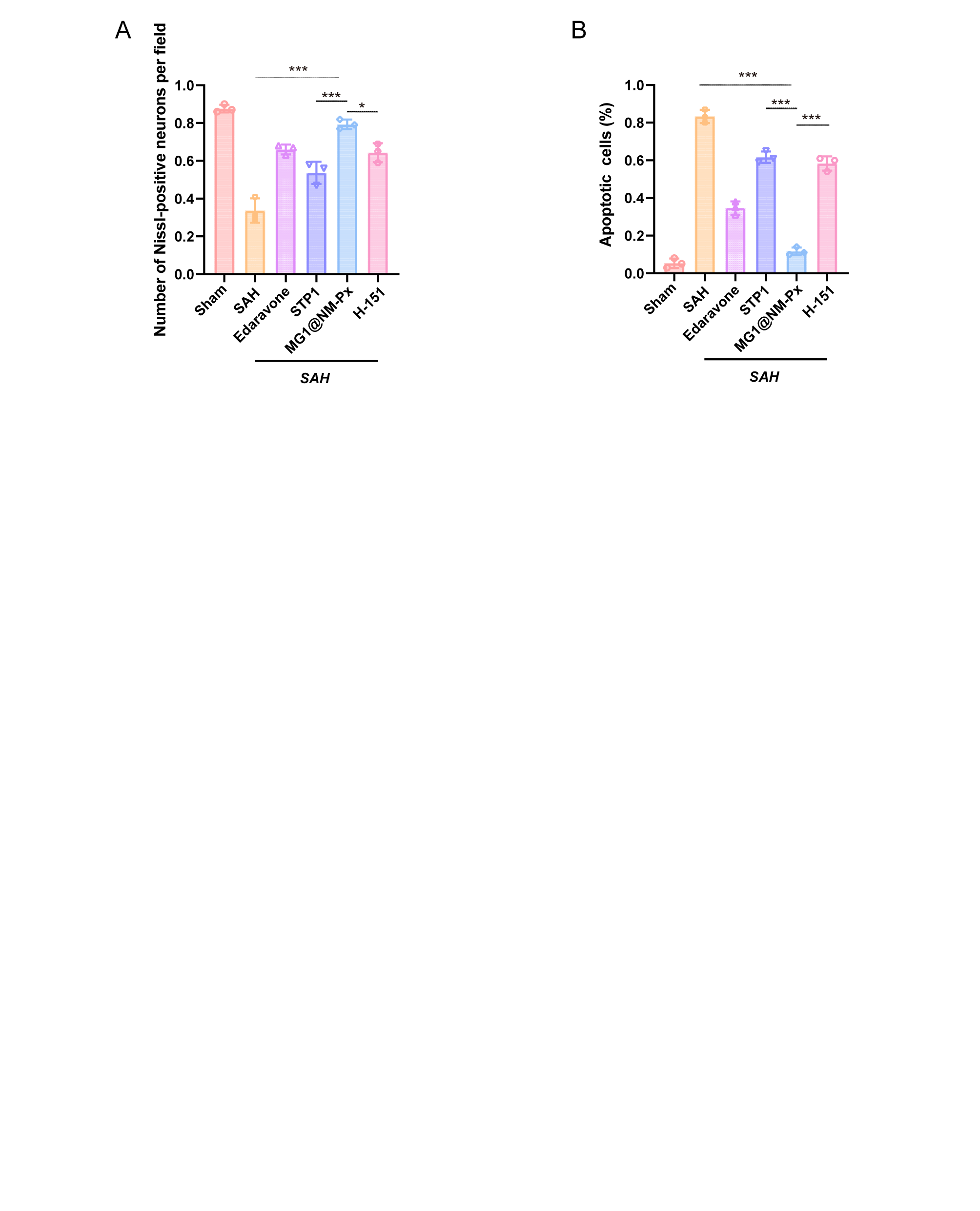


**Figure S27. Quantitative analysis of Nissl and TUNEL staining corresponding to Figure 6G and H.** (A) Quantification of Nissl-positive neurons per field in hippocampal sections from Sham, SAH, SAH + Edaravone, SAH + STP1, SAH + MG1@NM-Px, and SAH + H-151 groups. (B) Quantification of apoptotic cells based on TUNEL staining in brain sections from the indicated groups. MG1@NM-Px treatment significantly preserved Nissl-positive neurons and reduced apoptotic cell death after SAH compared with the untreated SAH group and other therapeutic interventions. Data are presented as mean ± SEM (n = 3 mice per group). Statistical significance was determined by one-way ANOVA followed by Tukey’s multiple-comparison test. **p* < 0.05, ****p* < 0.001.


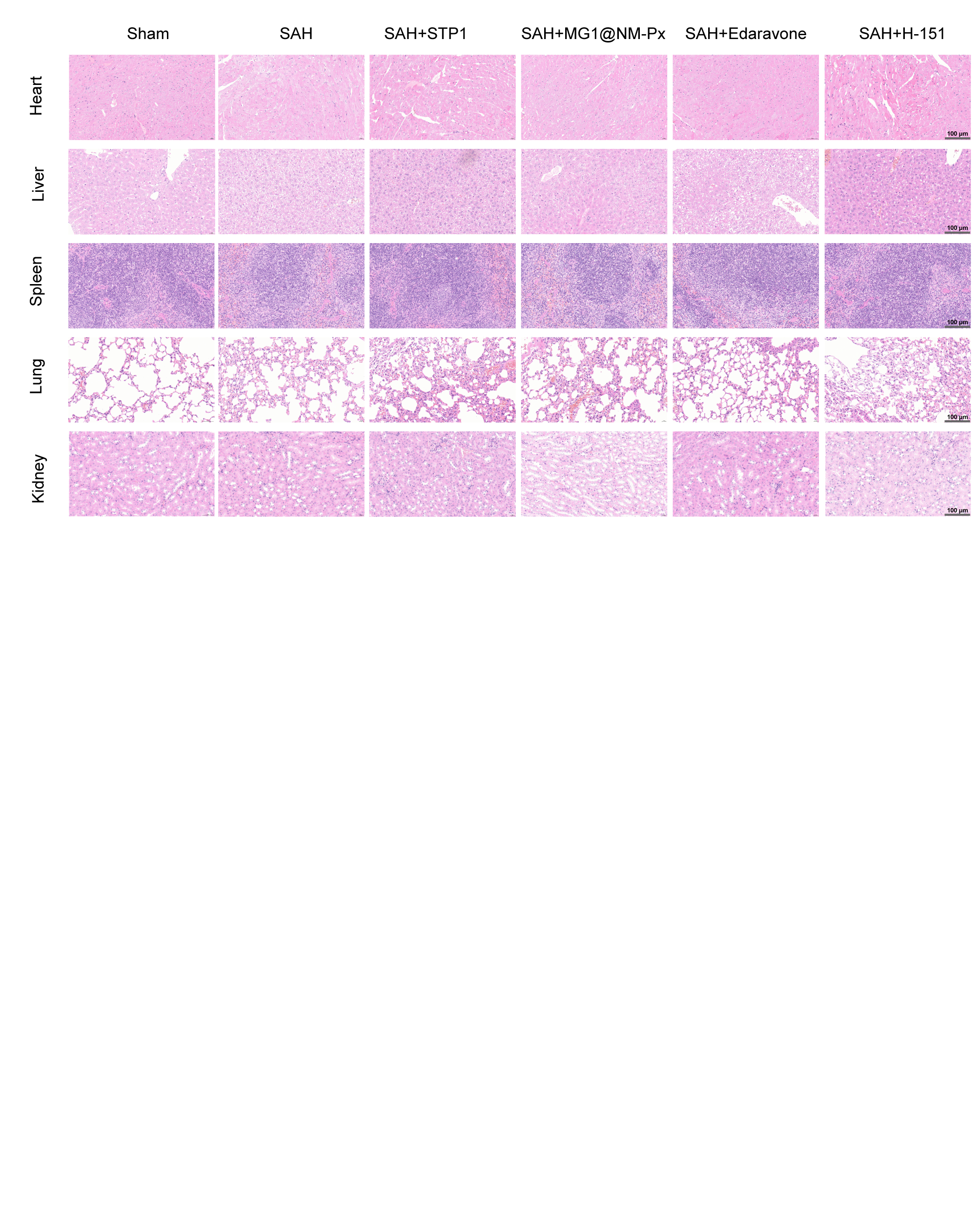
**Figure S28. Histological evaluation of major organs after different treatments.** Representative hematoxylin and eosin (H&E) staining images of major organs, including heart, liver, spleen, lung, and kidney, collected from mice in the Sham, SAH, SAH+STP1, SAH+MG1@NM-Px, SAH+Edaravone, and SAH+H151 groups. Scale bar: 100 μm.


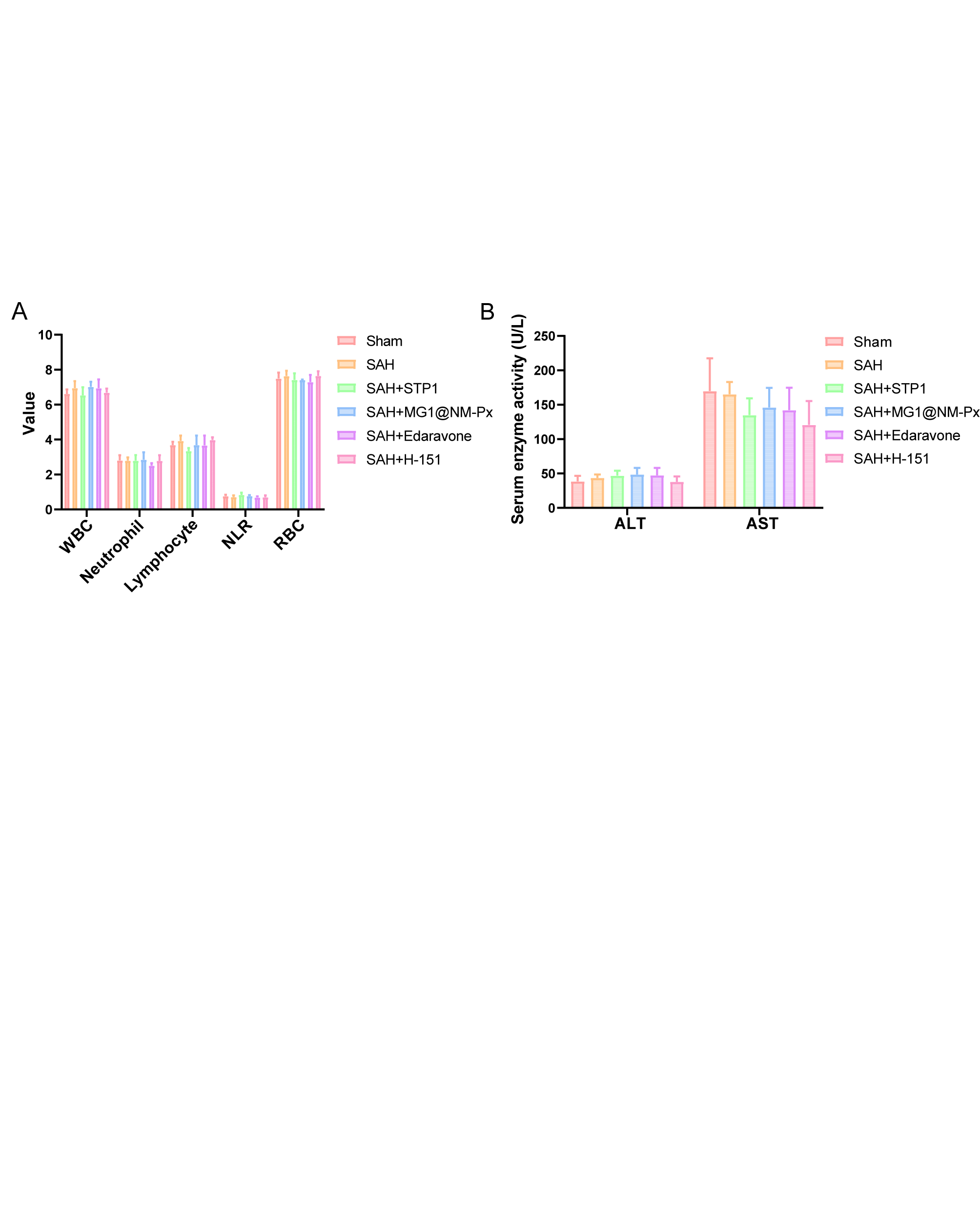
**Figure S29. Hematological and serum biochemical evaluation of systemic biosafety in mice after different treatments.** (A) Routine hematological parameters, including white blood cell count (WBC), neutrophil count, lymphocyte count, neutrophil-to-lymphocyte ratio (NLR), and red blood cell count (RBC), were measured in mice from the Sham, SAH, SAH+STP1, SAH+MG1@NM-Px, SAH+Edaravone, and SAH+H-151 groups. NLR was calculated as the ratio of neutrophil count to lymphocyte count. WBC, neutrophil, and lymphocyte counts are expressed as ×10⁹/L; RBC is expressed as ×10¹²/L; NLR is unitless. (B) Serum biochemical analysis of liver function indicators, including alanine aminotransferase (ALT) and aspartate aminotransferase (AST), in each group. ALT and AST are expressed as U/L. Data are presented as mean ± SEM, n = 3 per group. Statistical comparisons were performed using one-way ANOVA. ns, not significant.

**Table S1. Pharmacokinetic parameters of STP1 after intravenous administration of MG1@NM-Px.**

| **Sample** | **Cmax (mg/L)** | **CLz (L/min/kg)** | **AUC0–t (mg/L·min)** | **AUC0–∞ (mg/L·min)** | **MRT0–t (min)** | **t1/2 (min)** |
| --- | --- | --- | --- | --- | --- | --- |
| MG1@NM-Px | 0.162 ± 0.008 | 0.0217 ± 0.0004 | 19.37 ± 0.29 | 19.86 ± 0.34 | 563.93 ± 6.23 | 537.18 ± 31.17 |

Table note: Data are presented as mean ± SEM (n = 3 mice). MG1@NM-Px was intravenously administered at an STP1-equivalent dose of 8.6 μg per 20 g mouse, corresponding to 0.43 mg/kg. Pharmacokinetic parameters were calculated by non-compartmental analysis based on blood STP1 concentrations from 0.083 to 48 h after injection. Tmax was 5.0 min.

**Table S2. Brain-to-blood distribution of STP1 at 48 h after MG1@NM-Px administration**

| **Sample** | **Brain STP1 concentration (ng/g tissue)** | **Blood STP1 concentration (ng/mL)** | **Brain-to-blood ratio (mL/g)** |
| --- | --- | --- | --- |
| MG1@NM-Px | 4.73 ± 1.03 | 0.63 ± 0.08 | 7.35 ± 0.74 |

Table note: Data are presented as mean ± SEM (n = 3 mice). Brain and blood samples were collected at 48 h after intravenous administration of MG1@NM-Px. The brain-to-blood ratio was calculated as Cbrain/Cblood, where Cbrain was expressed as ng/g tissue and Cblood was expressed as ng/mL.
